# ARTS and small-molecule ARTS mimetics upregulate p53 levels by promoting the degradation of XIAP

**DOI:** 10.1007/s10495-024-01957-2

**Published:** 2024-04-29

**Authors:** Ruqaia Abbas, Oliver Hartmann, Dorin Theodora Asiss, Rabab Abbas, Julia Kagan, Hyoung-Tae Kim, Moshe Oren, Markus Diefenbacher, Amir Orian, Sarit Larisch

**Affiliations:** 1 Cell Death and Cancer Research Laboratory, Department of Human Biology and Medical Sciences, University of Haifa, 31905 Haifa, Israel; 2 Comprehensive Pneumology Center (CPC)/Institute of Lung Health and Immunity (LHI), Helmholtz Munich, Munich, Germany; 3 Shaperon Inc. Seoul, Seoul, Republic of Korea; 4 Department of Molecular Cell Biology, Weizmann Institute of Science, 7610001 Rehovot, Israel; 5 Rappaport Research Institute and Faculty of Medicine, Technion Integrative Cancer Center Technion- IIT, 3109610 Haifa, Israel; 6 Ludwig-Maximilian-Universität München (LMU), Munich, Germany; 7 German Cancer Consortium (DKTK), LMU, Munich, Germany

**Keywords:** Apoptosis, XIAP, p53, ARTS, Small-molecules

## Abstract

Mutations resulting in decreased activity of p53 tumor suppressor protein promote tumorigenesis. P53 protein levels are tightly regulated through the Ubiquitin Proteasome System (UPS). Several E3 ligases were shown to regulate p53 stability, including MDM2. Here we report that the ubiquitin E3 ligase XIAP (X-linked Inhibitors of Apoptosis) is a direct ligase for p53 and describe a novel approach for modulating the levels of p53 by targeting the XIAP pathway. Using in vivo (live-cell) and in vitro (cell-free reconstituted system) ubiquitylation assays, we show that the XIAP-antagonist ARTS regulates the levels of p53 by promoting the degradation of XIAP. XIAP directly binds and ubiquitylates p53. In apoptotic cells, ARTS inhibits the ubiquitylation of p53 by antagonizing XIAP. XIAP knockout MEFs express higher p53 protein levels compared to wild-type MEFs. Computational screen for small molecules with high affinity to the ARTS-binding site within XIAP identified a small-molecule ARTS-mimetic, B3. This compound stimulates apoptosis in a wide range of cancer cells but not normal PBMC (Peripheral Blood Mononuclear Cells**)**. Like ARTS, the B3 compound binds to XIAP and promotes its degradation via the UPS. B3 binding to XIAP stabilizes p53 by disrupting its interaction with XIAP. These results reveal a novel mechanism by which ARTS and p53 regulate each other through an amplification loop to promote apoptosis. Finally, these data suggest that targeting the ARTS binding pocket in XIAP can be used to increase p53 levels as a new strategy for developing anti-cancer therapeutics.

**Supplementary Information:**

The online version contains supplementary material available at 10.1007/s10495-024-01957-2.

## Introduction

Apoptosis is a form of programmed cell death essential for embryonic development and tissue homeostasis. Abnormal regulation of apoptosis leads to various human diseases, including neurodegeneration and cancer [1–3]. The apoptotic pathway activates caspases (cysteine–aspartic proteases) through the cleavage of their inactive zymogens. These enzymes act in a cascade that culminates in the cleavage of multiple cellular proteins, resulting in the disassembly of the cells [4, 5]. In living cells, caspases are kept in check by the X-linked inhibitor of apoptosis (XIAP) E3 ubiquitin ligase [6, 7]. XIAP contains three Baculoviral IAP repeats (BIR), which serve as protein–protein interaction domains [7–10]. XIAP-BIR3 binds and inhibits caspase-9, while BIR2 and a segment N-terminal to it are responsible for binding and inhibiting caspase-3 and -7 [7–10]. XIAP has a ubiquitin-associated (UBA) domain, which enables the binding of polyubiquitin conjugates, and a RING domain responsible for E3-ligase activity [11–13]. Upon induction of apoptosis, XIAP-mediated inhibition of caspases is counteracted by the IAP-antagonists SMAC, OMI/HTRA and ARTS [14–20].

ARTS (sept4-i2) is a splice variant derived from the *Septin4* gene, the only splice variant that regulates apoptosis [21]. ARTS is a pro-apoptotic and tumor suppressor protein localized on the mitochondrial outer membrane (MOM) [15]. Overexpression of ARTS is sufficient to induce apoptosis in various cancer cell lines, and it increases the sensitivity of cells to a wide variety of apoptotic stimuli [19, 22]. Human and mouse studies have shown that ARTS functions as a tumor suppressor protein and physiological antagonist of XIAP in vivo [23, 24]. ARTS expression is lost in more than 70% of acute lymphoblastic leukemia (ALL) patients and 50% of lymphoma patients and in a significant fraction of hepatocellular carcinoma (HCC) patients [23, 24]. *Sept4*/ARTS deficient mice have elevated XIAP levels and increased tumor incidence [24–27]. Upon apoptotic stimuli, ARTS rapidly translocates to the cytosol to bind and antagonize XIAP [14, 15]. Unlike other IAP antagonists, ARTS lacks the canonical IAP-Binding Motif (IBM) and instead binds XIAP-BIR3 via its unique C-terminal sequence [14, 28–31]. Furthermore, ARTS binds to a distinct sequence within XIAP-BIR3, which is different from the SMAC binding sequence within XIAP-BIR3 [28, 32]. ARTS is the only IAP-antagonist that promotes the degradation of XIAP through the ubiquitin–proteasome system (UPS) [28, 33, 34]. The direct binding of ARTS to XIAP results in the degradation of the latter and enables the release of active caspases from XIAP [15].

The p53 tumor suppressor and pro-apoptotic protein functions primarily as a sequence-specific transcription factor [35, 36]. Through binding to genomic DNA sequences known as a p53 binding sites or p53 response elements [37, 38], p53 controls the transcription of target genes that regulate various cellular processes, including cell cycle arrest, DNA damage repair, senescence, and apoptosis [35, 36, 39]. p53 promotes apoptosis through direct interaction with pro- and anti-apoptotic proteins [40–42]. p53 induces the transcription of the death receptor 5 (DR5), TNFR1, and Fas, which results in the activation of caspase-8 [43, 44]. In addition, p53 can induce transcription of pro-apoptotic Bcl-2 family proteins, such as BAX, PUMA, BAD, BID, BAK, and NOXA [45–48]. The induction of BID, BAK, and BAX promotes the permeabilization of the outer mitochondrial membrane and amplifies the caspase activation process [39, 49–51]. p53 also stimulates the transcription of ARTS, which relieves caspases from their inhibition by XIAP, leading to the cleavage of BID and MOMP [15, 52]. The levels of p53 are tightly controlled by the UPS [53–57]. The main negative regulator of p53 levels is the E3 ligase MDM2 [56, 58–61]. Many cancers escape apoptosis by reducing p53 levels via overexpressing its E3-ligases [62]. Moreover, mutations in the *TP53* gene impair p53’s tumor suppressor activity and may sometimes even confer oncogenic properties upon the mutant p53 [63–66]. Intense efforts have been made to develop anti-cancer drugs that can restore normal p53 activity, but so far, clinical results have been disappointing [37, 65, 67, 68].

Here, we describe the identification of XIAP as a distinct E3 ligase of p53 and show that ARTS upregulates p53 by antagonizing XIAP. In addition, our results suggest that ARTS and p53 regulate each other in an amplification loop. Moreover, we describe a small-molecule ARTS-mimetic, B3, which directly binds to the ARTS binding pocket in XIAP-BIR3. Similar to ARTS, B3 promotes apoptosis by downregulating XIAP levels, which in turn causes upregulation of p53 and apoptosis.

## Methods and materials

### Cell line culture

MEFs WT and A375 cells were grown in complete DMEM medium (1% sodium pyruvate, 1% L-glutamate, 1% non-essential amino acids, 1% Pen-Strep, 10% fetal bovine/calf serum, and 0.1% β-mercaptoethanol).

HCT 116 WT cells were grown in complete McCoy’s medium (1% sodium pyruvate, 1% l-glutamate, 1% Pen-Strep, and 10% fetal bovine/calf serum).

A549 cells were grown in DMEM/F12 complete medium (1% sodium pyruvate, 1% L-glutamate, 1% Pen-Strep, and 10% fetal bovine serum).

All cell lines were mycoplasma free and kept under passage 10.

### SDS-PAGE and western blot analysis

Cells were lysed in whole-cell extract buffer [25 mM HEPES, pH 7.7, 0.3 M NaCl, 1.5 mM MgCl2, 0.2 mM EDTA, 0.1% Triton X-100, 100 μg/ml phenylmethylsulfonyl fluoride (PMSF) and protease inhibitor cocktail (Roche, 1:100 dilution)] and placed on ice for 30 min (vortexing once after 15 min). After 30 min, the samples were centrifuged at 13,000 × g for 10 min at 4 °C. The supernatants containing total protein were analyzed for protein concentration using the Bio-Rad Protein Assay Dye Reagent Concentrate Kit. Proteins (40–60 µg) were separated by sodium dodecyl sulfate–polyacrylamide gel electrophoresis (SDS-PAGE; 12% or 7.5%), followed by transfer to a nitrocellulose membrane. Membranes were blocked with 5% (w/v) non-fat dried skimmed milk powder in PBS supplemented with 0.05% Tween-20 (PBS-T) for 1 h at RT. Next, primary antibodies were added at 4 °C overnight or for 2 h at room temperature. Membranes were then incubated with the secondary antibody for 1 h at RT and washed three times for 15 min each with PBS-T. Western Bright ECL (Advansta) was added to the membrane for 30–60 s and analyzed using the Image Quant LAS-4000 (GE Healthcare Life Sciences) and Image Quant LAS-4000 software (GE Healthcare Life Sciences). Densitometry of proteins levels were determined by Image studio lite v5.2. Protein levels were normalized to the control (DMSO) levels.

### Treatments and reagents

Cells were pre-incubated with 20 µM MG-132 (APExBIO cat#A2585) or Bortezomib (abcam cat#ab142123) for 4 or 6h. 20µM of B3 was added during the last 2 or 5 h of incubation with the proteasome inhibitor. The cells were treated with 200µM µM Etoposide (abcam cat#ab120227) for three hours. Cells were treated with 200ng/ml Nocodazole (sigma cat#m1404).

### Co-immunoprecipitation

Cells were harvested and lysed with radioimmunoprecipitation assay (RIPA) buffer (Tris–HCl pH 7.5 50 mM, NaCl 150 mM, NP-40 (Igepal) 0.3%) containing protease inhibitor cocktail (Complete, Roche) and 100 μg/ml PMSF. Antibodies were used at 5 µg per 1000 µg protein and incubated overnight, rotating at 4 °C. The next day, agarose beads conjugated to protein A/G (Santa Cruz Biotechnology) were added for 4 h with rotation at 4 °C. samples were centrifuged at 4 °C for 5 min and washed five times with RIPA buffer. Proteins were eluted from the beads after 10 min of boiling in sample buffer and separated on a 12% SDS-PAGE gel, followed by western blot analysis.

### Antibodies

ARTS, a mouse monoclonal anti-ARTS antibody, specifically targeting the unique C-terminal sequence of ARTS (but not other *septin4* splice variants) at a dilution of 1:1000 (Sigma A4471).

XIAP, mouse monoclonal anti-XIAP antibody (BD cat#610,717) at a dilution of 1:4000.

XIAP, mouse monoclonal anti-XIAP antibody (Santa cruz ac-55550) at a dilution of 1:1000.

XIAP and rabbit monoclonal anti-XIAP antibody (Cell Signaling cat#CS14334) at a dilution of 1:3000.

Actin was used as the mouse monoclonal anti-actin antibody (ImmunoTM cat#08691002) at a dilution of 1:50,000.

Cleaved PARP (cl.PARP) and rabbit monoclonal anti-cl. PARP antibody (Cell Signaling Technology, #CS5625) at a dilution of 1:2000.

Tubulin, a monoclonal rat anti-tubulin antibody (Abcam cat#YOL1/34), was used at a dilution of 1:6000.

p53, rabbit monoclonal anti- p53 antibody (Cell Signaling cat#CS32532) at a dilution of 1:4000.

p53 and mouse monoclonal anti- p53 antibody (Cell Signaling cat#CS2524) were used for immunoprecipitation.

p53, mouse monoclonal anti- p53 antibody (Santa Cruz DO-1 cat#SC-126) at a dilution of 1:1000.

p53, goat polyclonal anti-p53 antibody (R&D cat#AF1355) at a dilution of 1:6000.

### Plasmids and Transfections reagents

myc-ARTS, pCMV-ARTS, HA-Ubiquitin, myc-XIAP. The following reagents were used according to the manufacturer’s instructions for transient transfections: Transit-X2 (Mirus) and PolyJet (SL100688).

### In vivo ubiquitylation assay (in cell culture)

Indicated cells were transiently transfected with a Ub-HA (ubiquitin-tagged with HA) construct and treated with a proteasome inhibitor (Bortezomib or MG-132, at 20 µM for 4h or 6 h). After 1h or 2h of incubation with proteasome inhibitor, B3 (20 μM in DMSO) or DMSO was added to the medium for an additional 2h or 5 h. Subsequently the cells were harvested and lysed using RIPA buffer [50 mM Tris–HCl pH 7.5, 150 mM NaCl, 0.3%NP-40 (Igepal)] containing protease inhibitor cocktail (Complete, Roche), 100 μg/ml PMSF, 5 mM N-ethylmaleimide, and 5 mM iodoacetamide to preserve the ubiquitin chains. After 15 min of centrifugation (10,000 × g, 4 °C), the supernatant was transferred to a clean Eppendorf tube. Endogenous P53 was recovered from the extract by immunoprecipitation (CS2524) with 1:500 antibody per 1000 µg protein. p53 and its poly ubiquitylated forms of p53 were detected using an anti-p53 antibody (DO-1, sc-126). Densitometry analysis was done using Image studio lite v5.2. The levels of ubiquitylated XIAP and p53 were normalized to the levels of the un-ubiquitylated XIAP and p53 proteins, respectively.

### In vitro ubiquitylation assay

In vitro ubiquitylation in a fully reconstituted system was performed using bacterially expressed His-ARTS was purified using fast protein liquid chromatography (FPLC) and bacterially expressed GST-XIAP was purified using glutathione beads. p53 recombinant protein was purchased from R&D Systems (cat#SP-450). In vitro ubiquitylation contained recombinant p53 (0.2µg) and GST-XIAP (0.2µg). E1, UbcH5b, ubiquitin (Ub), and E3 in conjugation buffer (40mM Tris [pH 7.5], 5mM MgCl_2_, Ubiquitin 5μg, and 10mM DTT) containing 2mM ATP_γ_s and Ubiquitin aldehyde incubated at 37C° for 1 h. ARTS (0.2, 0.4, 0.8µM) and B3 (20 and 40µM) were added to the reaction mix ( Subsequently proteins were resolved over SDS-PAGE and the indicated antibodies.

### Bimolecular fluorescence complementation assay (BiFC)

The Split-Venus BiFC system was used to evaluate close proximity, indicating possible direct binding between pairs of proteins. The XIAP, ARTS and p53 proteins were fused either to the N-terminal part of Venus-YFP (yellow fluorescence protein) (VN) or the C-terminal domain (VC). All Venus fragments were fused to the N-terminal sequences of the XIAP, ARTS and p53 proteins. The Jun and bFos pair was used as a positive control (p.c.) and the Jun and bFosdeltaZIP pairs were used as negative controls (n.c.). A vector encoding dsRed was used as the transfection efficiency marker. Venus (BiFC) and dsRed signals in cells were quantified in a FACSCantoII flow cytometer (BD Biosciences, San Jose, CA, USA) equipped with an argon laser emitting at 488 nm. Analysis was restricted to live cells. Results were analyzed using FACSDIVA software (BD Biosciences). To normalize the results per transfection efficiency, the mean fluorescence intensities of the BiFC complexes were normalized to the mean fluorescence intensity of dsRED. The ratio between YFP and Red fluorescence was calculated for each time point. At least 10,000 cells were analyzed in each experiment.

### Duolink- proximity ligation assay (PLA)

To detect the interaction between XIAP and p53, we used the Duolink® In Situ PLA® kit (DUO92101, Sigma-Aldrich). The Duolink proximity ligation assay was performed following the manufacturer’s method. Fluorescence images were obtained under a confocal laser scanning microscope (Zeiss LSM 700) using a 63 × oil objective lens.

### MST binding assays

Microscale thermophoresis (MST) binding assays were performed by CreLux, a WuXi AppTech company in Germany, using recombinant ARTS and XIAP proteins. To perform experiments with untagged XIAP, a fluorescent label (NT650) was covalently attached to the protein (maleimide coupling). Labeling was performed in buffer containing 50 mM HEPES (pH 7.0), 150 mM NaCl, and 0.005% Tween-20.

### Computational screen

Three hundred thousand commercially available molecules were selected from a set of ~ 3 million and screened using LeadIT and SeeSAR software suits from BioSolveIT. This computational screen identified compounds with predicted binding affinities in the micro-molar to Nano-molar range, as assessed by the HYDE scoring function. The 100 top-ranked molecules with the best docking scores were identified. The ARTS-unique binding site in XIAP-BIR3 was extrapolated by analyzing XIAP-SMAC crystal structures from the PDB and our data, as described by Bornstein et al.

### Preparation of B3 stock and work solution

The B3 small molecule (MW 406.43 gr/mol as powder, SMILES: (CC22H22N4O4) was purchased from eMolecules, Inc., eMolecule ID:30,500,827 (Supplier InterBioScreen STOCK 6S-95262). B3 was dissolved in dimethyl sulfoxide (DMSO) to a stock solution of 30mM, followed by intensive pipetting and centrifugation at 300 × g for 30 s. Next, the B3 suspension was incubated in a 37 °C bath for 1 min, mixed thoroughly by pipetting, and spun down again. B3 stock solution was aliquoted in Eppendorf tubes (7–10 µL/tube) and stored at -80 °C. Aliquots were used only once to avoid freezing and thawing the compound. Before use, the B3 aliquot was thawed, spun down (using the same settings), and mixed by gently tapping the lower part of the Eppendorf tube. Next, the compound solution was diluted 1:100 in a warm complete medium in a 15 ml conical tube to a concentration of 0.3mM and mixed well by tilting the closed vial up and down (do not vortex). The B3 solution was then diluted again to the desired final concentration (5–40 µM) and added to the cells.

### Statistical analysis

All graphs were generated using the PRISM software. Significance was evaluated using two-tailed, unpaired T-test, one-way analysis of variance (ANOVA) or two-way ANOVA. Statistical significance is denoted by *, **, or *** to indicate *P* < 0.05, *P* < 0.001, or *P* < 0.0001, respectively.

## Results

### p53 induces the transcription of ARTS in response to DNA damage

**Fig. 1:**
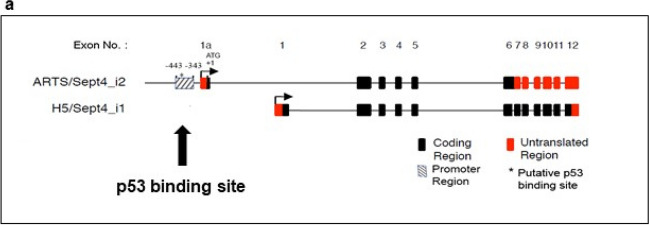
**a. Scheme of the putative p53 binding site in ARTS promoter sequences.** Scheme of *Sept4* gene. The *sept4* gene encodes two main splice variants, Sept4_i1 (PNUTL2, H5) and Sept4_i2 (ARTS). The scheme shows the location of a putative p53 binding site at the ARTS promoter located between nucleotides -391 to -351 upstream of the ARTS TSS

P53 induces apoptosis through the transcriptional induction of target genes [35, 36]. Sequence-specific DNA binding of p53 is a prerequisite for trans-activating target genes [35–37, 69]. ARTS is a splice variant of the human Sept4 gene located on chromosome 17q22-23 [70]. Differential splicing of Sept4 mRNA generates two isoforms, Sept4_i1 and ARTS (Sept4_i2) (Fig. 1a) [21]. Importantly, the mRNAs of both isoforms originate from two distinct transcription start sites (TSS), and the expression, tissue distribution, and function of ARTS and Sept4_i1 are distinct [70]. The proximal promoter gives rise to Sept4_i1, and a more distally located promoter is responsible for generating ARTS mRNA (Fig. 1a). Kostic and Shaw reported a putative p53-binding site in Sept4 [71]. Indeed, by using bioinformatics analysis, we identified a p53 binding motif between nucleotides -391 and -351 located upstream of the ARTS TSS (Fig. 1a). This motif contains two half-sites that strongly resemble the consensus DNA sequence sufficient for p53 binding [72]. Using a chromatin immunoprecipitation assay (ChIP) we found that binding of p53 to the ARTS-specific promoter sequences occurs within 5 min of UV irradiation in human colorectal carcinoma cells (HCT116 cells) (data not shown). Consistently, real-time PCR assays confirmed a similar rapid induction of ARTS transcription within 5 min following UV irradiation in WT HCT116 cells, with lower levels of ARTS mRNA in p53 knockout (KO) HCT116 cells (Supplementary Fig. 1). These observations are in agreement with published results showing that p53 acts as a transcriptional regulator of ARTS in response to DNA damage [52].

### ARTS affects p53 protein levels.

**Fig. 2:**
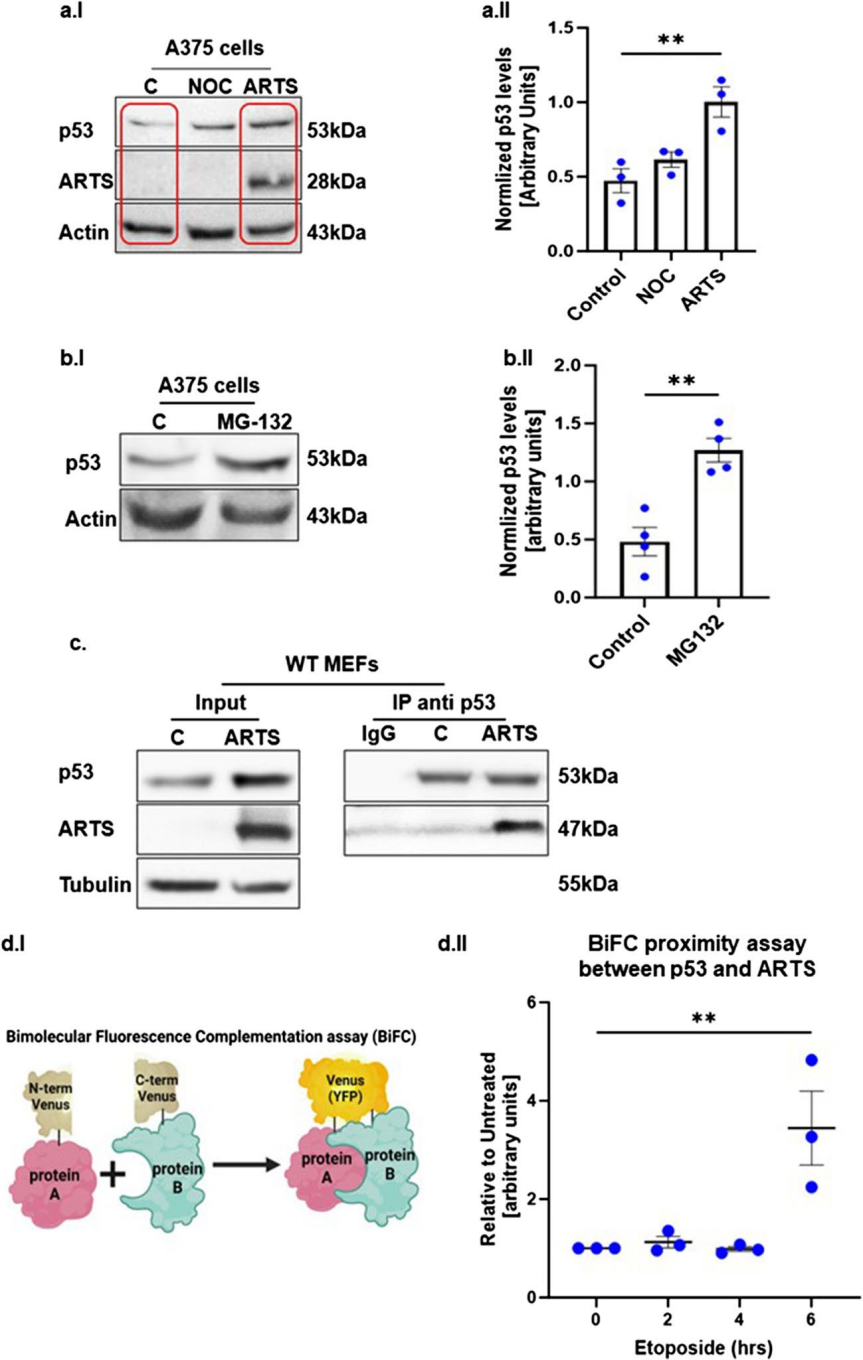
**a. ARTS increases the levels of p53 through proteasome-mediated degradation. a.I.** human melanoma A375 cells were transfected with ARTS and treated with 200ng/ml Nocodazole (NOC) for 1 h. Overexpression of ARTS upregulates the levels of p53 similar to treatment with Nocodazole. The panel labeled “C” represents control cells transfected with an empty vector **a. II.** Densitometry analysis of four biologically independent experimental repeats. **b. p53 protein levels are regulated through the UPS (Ubiquitin-Proteasome-System) in A375 cells. b.I.** A375 cells were treated with 20µM MG132 for 6 h, stabilized the p53 protein levels . “C” -control cells treated with DMSO **c.II.** Densitometry analysis of three independent experimental repeats **c. p53 binds to ARTS.** Co-immunoprecipitation experiments show that p53 binds to ARTS. **d. ARTS and p53 are in close proximity upon induction of apoptosis. d.I** Illustration of the BiFC assay. The fluorescent protein Venus (yellow fluorescent protein-YFP) is split into N- and C-terminal nonfluorescent fragments which are fused with the two proteins of interest, A, and B, respectively. If A and B interact directly, N-term YFP and C-term YFP will be in close proximity resulting in reconstitution of the fluorescent YFP **d.II** BiFC assays were performed on WT MEFs. Increased complex formation between ARTS-p53 is seen upon Etoposide treatment

The human melanoma cell line A375 has no detectable levels of ARTS. Here we show that introduction of exogenous ARTS alone was sufficient to upregulates p53 protein levels (Fig. 2bI, II). Next, we treated A375 cells with the proteasome inhibitor MG-132 and found that this caused a substantial accumulation of p53 protein (Fig. 2cI, II). This indicates that p53 levels are restricted by both ARTS and the UPS in these cells. To examine how ARTS regulates the levels of p53 we first tested if ARTS and p53 interact with each other. Results from bimolecular fluorescence complementation assays (BiFC) and immunoprecipitation assays show that ARTS and p53 are in close proximity with each other in etoposide-treated cells and presumably form a complex (Fig. 2d, eII).

### XIAP serves as an E3 ligase of p53

**Fig. 3:**
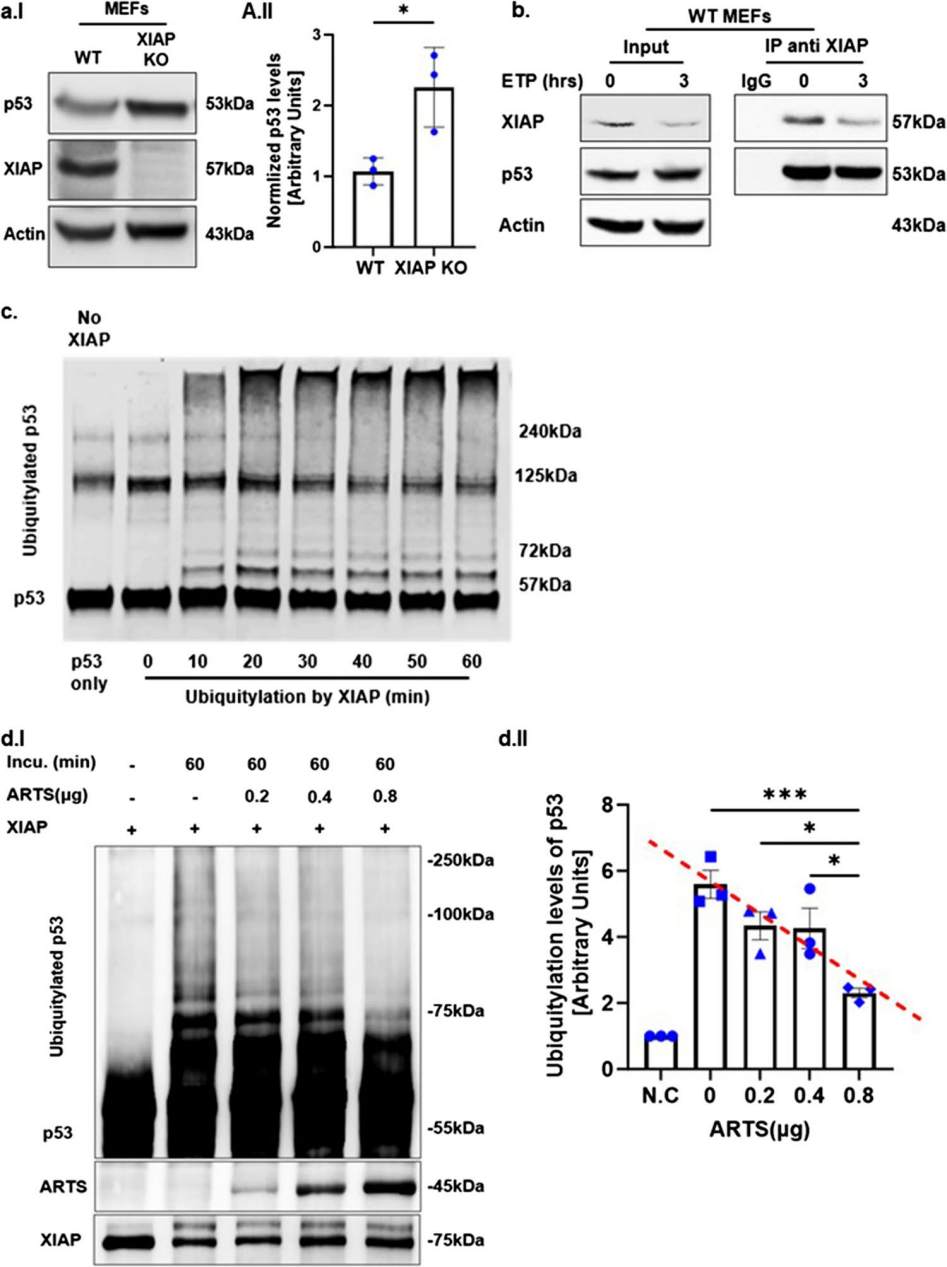
**XIAP is an E3 ubiquitin ligase of p53. a. Higher levels of p53 in XIAP KO MEFs compared to WT MEFs. a.I** Western blot analysis of ARTS and p53 in XIAP KO MEFs compared to their levels in WT MEFs. Actin serves as a loading control **a.II.** Densitometry analysis of three biologically independent experimental repeats. **b. XIAP binds p53.** Co-immunoprecipitation assays show that endogenous XIAP can bind to p53. **c. XIAP  promotes the ubiquitylation of p53. **In vitro*,* ubiquitylation assays were performed using a reconstituted ubiquitylation system, including recombinant XIAP and p53 proteins, E1 and UbcH5b as E2 for the indicated times. The first lane from left (p53 only) contained all components (including E1 and E2-UbcH5b) but no XIAP. XIAP ubiquitylates p53 as soon as 10 min after co-incubation. **d. ARTS inhibits p53 ubiquitylation by antagonizing XIAP**. **d.I.** In vitro ubiquitylation assays were performed using recombinant XIAP and p53 proteins. Increasing amounts of ARTS inhibited the ubiquitylation of p53 by XIAP in a dose dependent manner suggesting that ARTS impedes the complex between XIAP and p53 **d.II**. Densitometry analysis of three biologically independent experimental repeats

XIAP E3 ligase activity regulates the levels of several pro-apoptotic proteins, including ARTS and Bcl-2 [20, 56]. A combination of in vitro and in vivo ubiquitylation assays demonstrated that ARTS can bind to and induce the degradation XIAP, serving as its physiological antagonist [14, 15, 27–29, 33, 34, 56]. In particular, under apoptotic conditions, ARTS can promote the degradation of XIAP either by promoting its autoubiquitylation and degradation, or by acting as a scaffold for bringing XIAP into close proximity with the E3 ligase SIAH [28, 33, 73]. Therefore, we examined whether ARTS regulates the levels of p53 protein through its effect on XIAP. We found that mouse embryonal fibroblasts (MEFs) generated from XIAP knockout (KO) mice exhibited elevated levels of p53 compared to WT MEFs (Fig. 3a). Furthermore, immunoprecipitation assays confirmed that XIAP binds to p53 (Fig. 3b). Next, to assess whether XIAP serves as an E3 ligase of p53, in vitro ubiquitylation assays were performed using recombinant XIAP and p53 proteins, with UbcH5b as E2. Indeed, we show that XIAP ubiquitylates p53 as early as 10 min after co-incubation (Fig. 3c). We conclude that XIAP functions as an E3 ligase for p53. Moreover, in vitro ubiquitylation assays showed that ARTS reduces the ubiquitylation of p53 by XIAP in a dose-dependent manner (Fig. 3d). Thus, ARTS promotes the accumulation and upregulation p53 by inhibiting the ubiquitylation of p53 by XIAP (Fig. 3d).

### The small-molecule ARTS-mimetic B3 induces apoptosis by reducing XIAP and elevating p53 levels

**Fig. 4:**
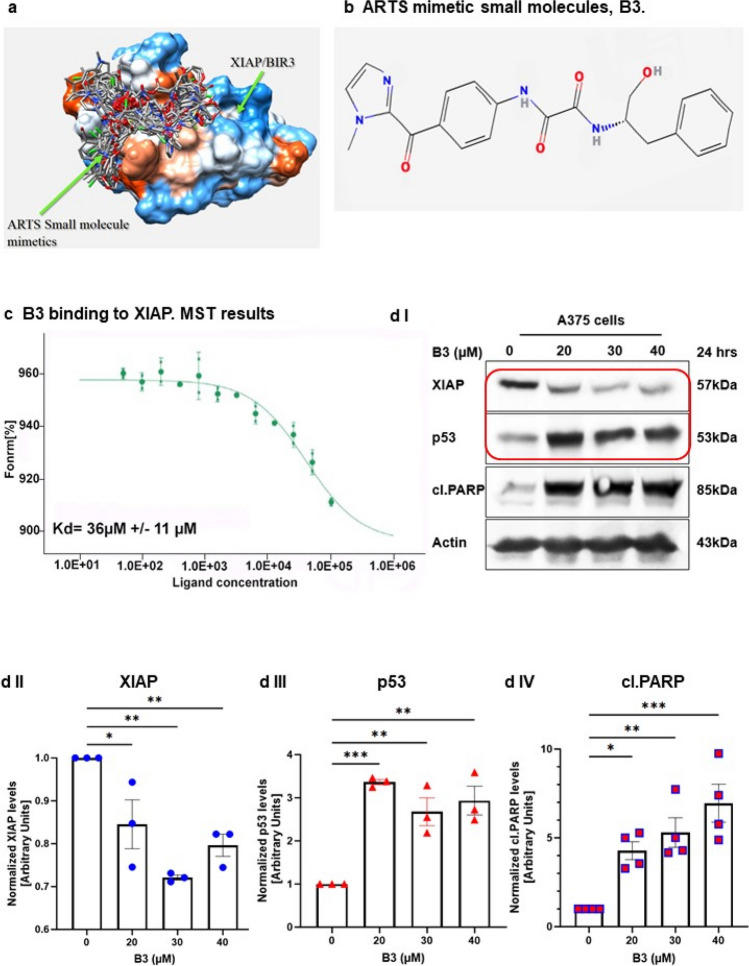

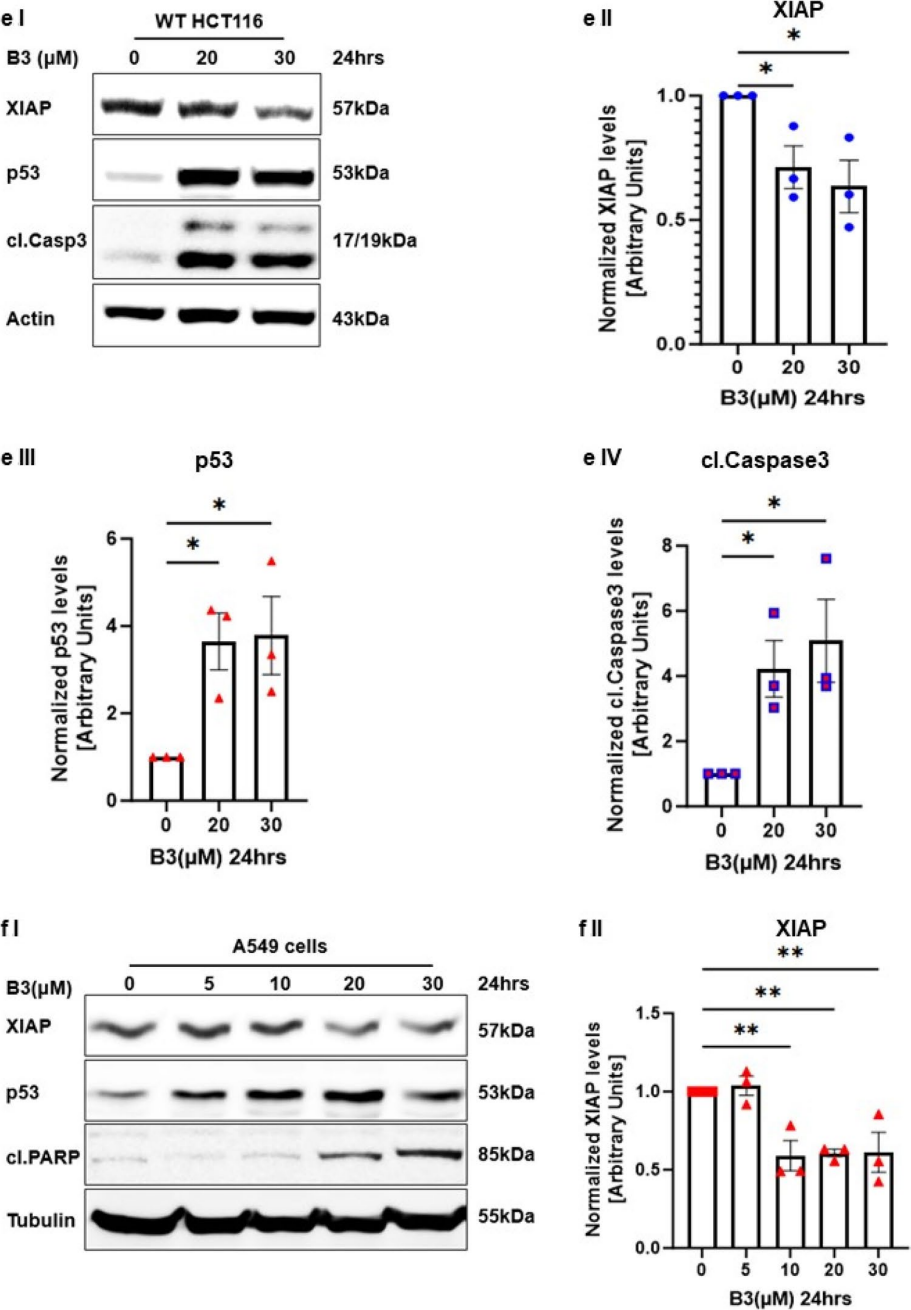

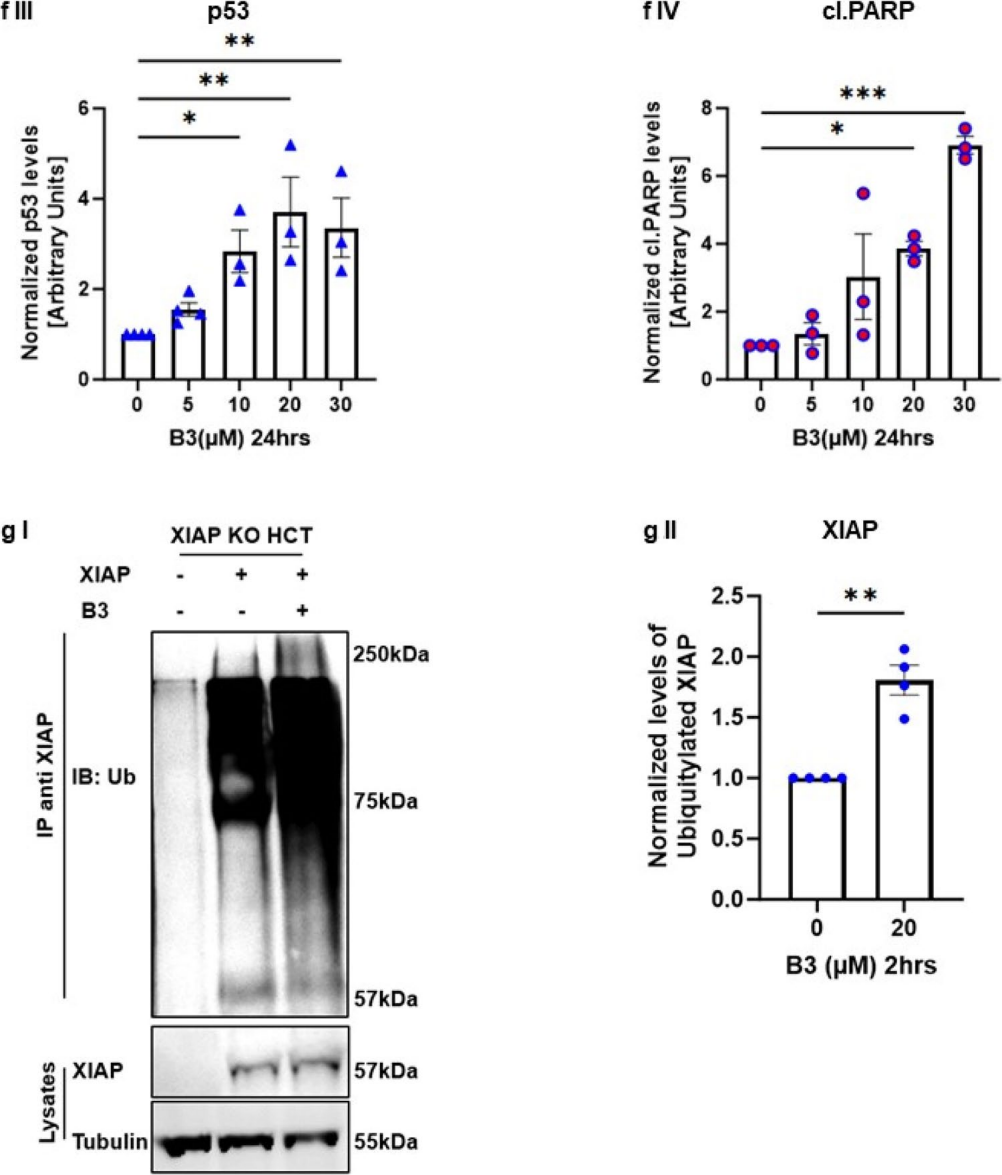
**Small-molecule ARTS-mimetics, B3, can reduce XIAP levels to promote apoptosis. a. Illustration of the 100 small molecules derived from the in “silico screen”.** An in silico screen was conducted by “BioSolveit” to look for ARTS-mimetic small molecules that fit into the distinct ARTS binding pocket in XIAP-BIR3. **b. B3 2D chemical structure**. **c. MST (microscale thermophoresis)** analysis of B3 binding to fluorescently labeled recombinant XIAP revealed a direct binding to XIAP-BIR3 with Kd of 36µM ± 11µM. **d-f. B3 decreases XIAP and upregulates p53 levels in a dose-dependent manner.** Western Blot analysis of the indicated proteins in three different cancer cells lines A375 cells (d) HCT116 cells (e) and A549 cells treated with various concentrartions of B3. In all these cells, treatment with B3 resulted in reduced levels of XIAP, increased levels of p53 and induction of apoptosis (as seen by increased levels of cleaved Caspase 3 or cleaved PARP). Densitometry analysis of three biologically independent experiments are shown for each protein and cell line. **g. B3 reduces the levels of XIAP through the UPS. g.I** XIAP ubiquitylation assays in cells were performed using XIAP knockout (KO) HCT116 cells. Ubiquitylation of XIAP transfected into XIAP KO cells was significantly increased upon treatment with the ARTS mimetic compound B3. **g.II** Densitometry analysis of three biologically independent experimental repeats

We previously reported a structure-based computational screen for small-molecule compounds that fit into the unique binding pocket for ARTS in XIAP-BIR3 [32]. Here, we characterized one of our top-ranked compounds, B3 (Fig. 4a). Using microscale thermophoresis (MST), we show that B3 binds to the XIAP-BIR3 domain with a Kd of 36μM (± 11μM) (Fig. 4c).

**Fig. 5:**
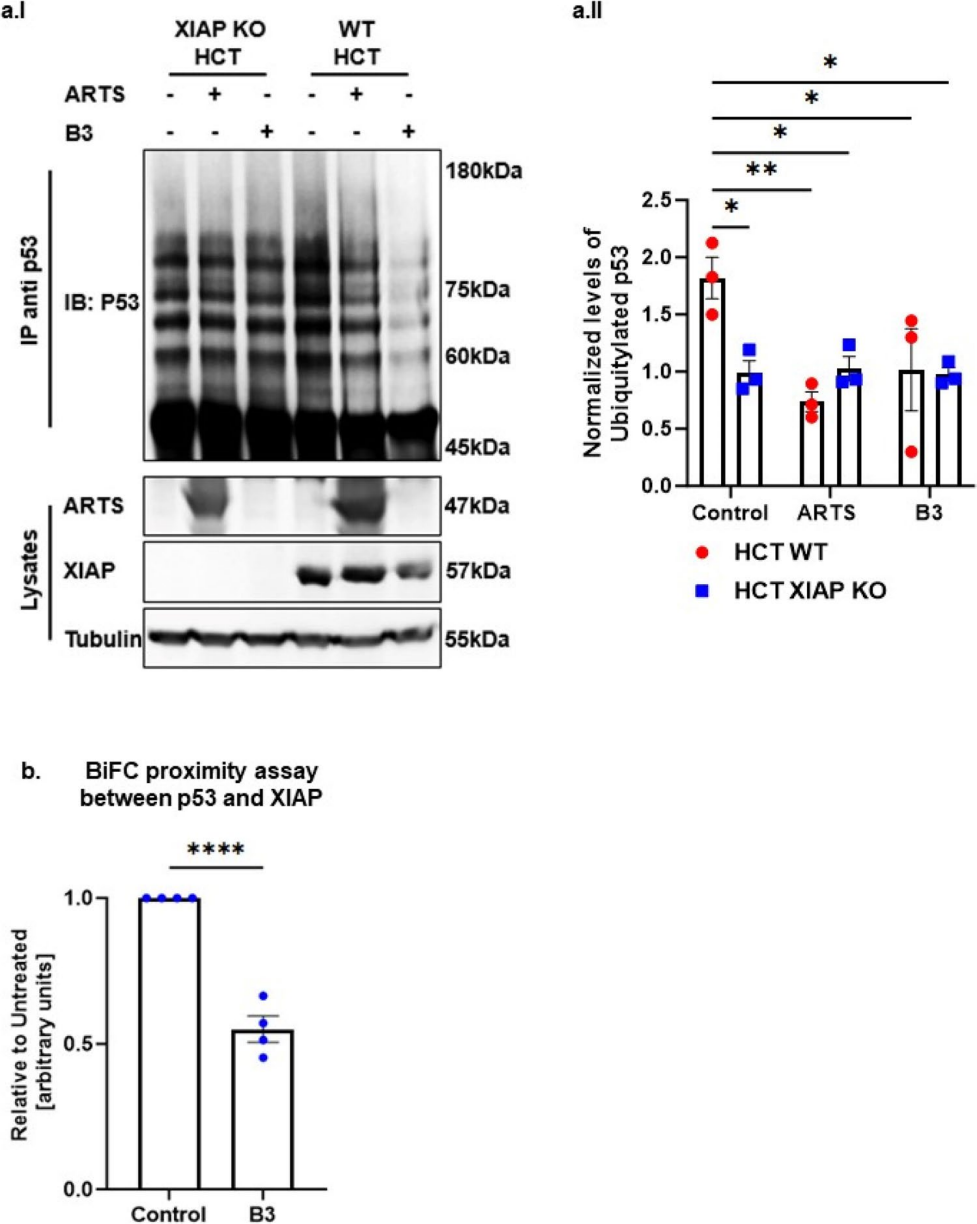

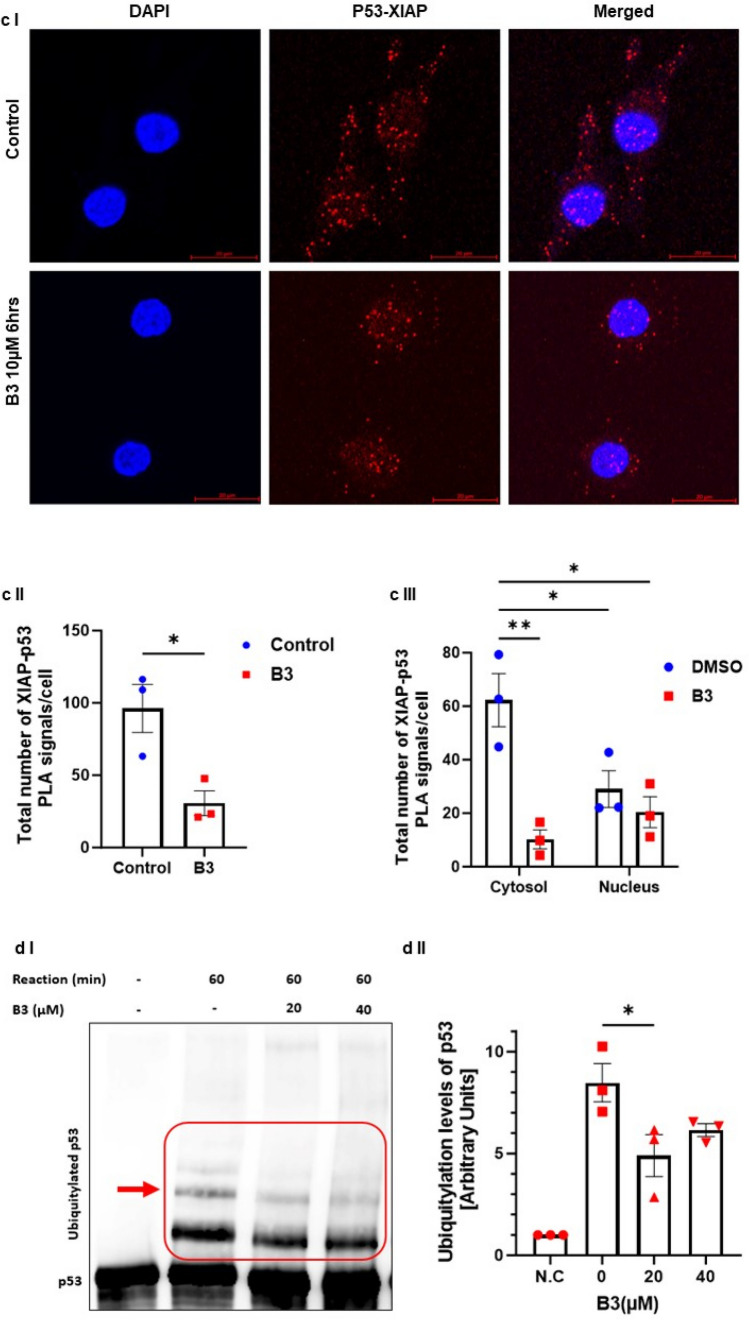
**Small molecule ARTS-mimetics B3 upregulate p53 levels by antagonizing XIAP. a. ARTS and the ARTS-mimetic B3 attenuate p53 ubiquitylation. a.I** Ubiquitylation assay of p53 was performed using WT and XIAP KO HCT116 cells; Western blot analysis shows that ARTS and ARTS-mimetic (B3) attenuate the ubiquitylation of p53. **a.II** Densitometry analysis of three independent experimental repeats **b. B3 disrupts the binding between XIAP and p53**. BiFC assay was performed on WT MEFs. Treatment with 20µM of B3 for 18h disrupted the formation of the XIAP-p53 complex. **c. B3 impedes the complex formation between p53 and XIAP.** In situ localization of the interaction between endogenous XIAP and p53. Interaction of endogenous XIAP and p53 was measured by Proximity Ligation Assay (PLA) in A375 cells treated with B3 10μM for 6h and DMSO as a control. **c.I.** Significant reduction in PLA signals indicates reduced interaction between XIAP and p53 following B3 treatment. The graph represents three independent experimental repeats **c.II.** B3 affects the complex formation between XIAP and p53 in the cytosol. The graph represents three independent experimental repeats. **d**.**B3 inhibits XIAP-mediated p53 ubiquitylation**. **d.I** In vitro ubiquitylation assays were performed by incubating recombinant XIAP and p53 with two concentrartions of B3. B3 downregulates p53 ubiquitylation by antagonizing XIAP. **d.II** Densitometry analysis of three biologically independent experimental repeats

To examine whether B3 can mimic the function of ARTS in antagonizing XIAP and upregulating p53, we tested the effect of B3 on protein levels of XIAP and p53 in three different cell types: A375, WT HCT and A549 cells (Fig. 4d, e, f respectively). Incubation with B3 led to a pronounced dose-dependent decrease in XIAP and increase in p53 protein levels, which culminated in induction of apoptosis (Fig. 4d, e, f). Importantly, B3 decreased the levels of XIAP by promoting its ubiquitylation (Fig. 4g). Next, we tested the effect of ARTS and B3 on the ubiquitylation of p53 in WT and XIAP knockout (KO) HCT116 cells. First, the XIAP KO HCT cells demonstrate lower levels of the ubiquitylated forms of p53 when compared to WT HCT cells, supporting the idea that XIAP acts as an E3 ligase for p53 (Fig. 5a). Moreover, both treatment with exogenous ARTS and with B3 inhibited the ubiquitylation of p53 in WT HCTs but not in XIAP KO. Thus, confirming again that ARTS and B3 affect p53 through their function on XIAP (Fig. 5a I, II). This provides a potential mechanism by which B3 can increase levels of p53 by inhibiting XIAP-mediated ubiquitylation. Furthermore, BiFC assays revealed that B3 disrupted the binding between p53 and XIAP (Fig. 5b and supplementary Fig. 3). To determine whether p53 and XIAP interact directly, we performed a Proximity Ligation Assay (PLA). This technique allows the identification of in situ interactions of two endogenous proteins. Our results indicate that in untreated cells XIAP and p53 co-localize mainly in the cytosol (Fig. 5b, c). Treatment with B3 significantly reduced the co-localization between XIAP and p53 (Fig. 5cI, II, III). Finally, results from in vitro ubiquitylation assays show that B3 can inhibit the ubiquitylation of p53 by XIAP (Fig. 5d). Together, these results suggest that ARTS as well as B3 can disrupt the binding between XIAP and p53 which culminates in stabilization of p53 and apoptosis.

**Fig. 6:**
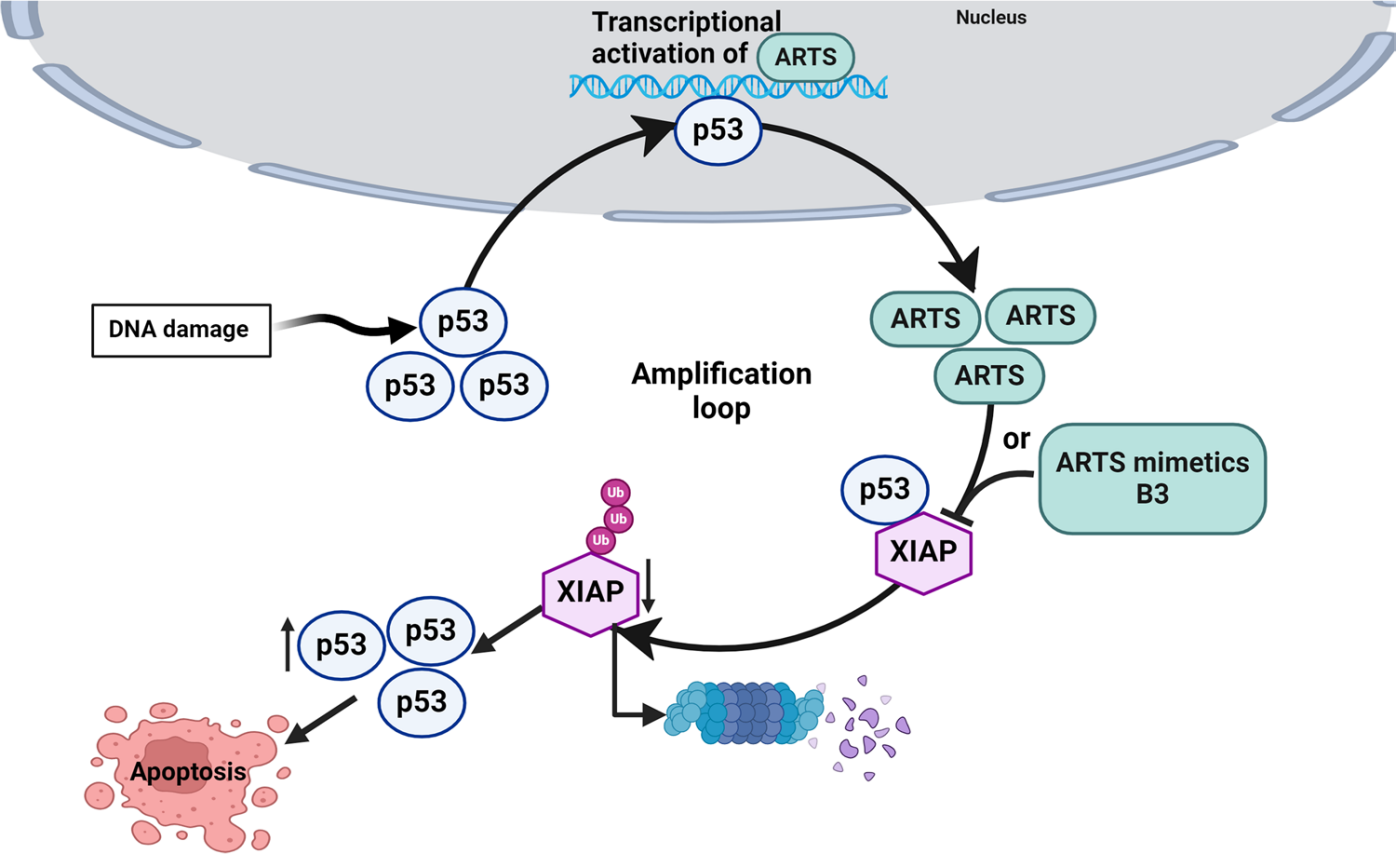
**Proposed model for the upregulation of p53 by ARTS and ARTS-mimetic small molecules.** Illustration of an amplification loop between p53 and ARTS. Upon stress signals, p53 levels increase, resulting in the transcription of its target genes, including ARTS. Upregulation of ARTS antagonizes XIAP by promoting XIAP degradation. This reduces ubiquitylation and stabilizes p53, causing an amplification loop that culminates in apoptosis. Similar to ARTS, B3 promotes the degradation of XIAP and increases the stability of p53 to promote apoptosis

Here, we demonstrate a distinct amplification loop mechanism by which ARTS and p53 mutually upregulate each other’s protein levels to promote apoptosis. Upon induction of apoptosis, p53 binds to specific binding elements within the ARTS promoter sequence and induce the transcription of ARTS. In turn, upregulation of ARTS induces the degradation of XIAP through the Ubiquitin–Proteasome System (UPS). XIAP serves as a direct E3-ligase of p53. Binding of both ARTS and ARTS mimetic small molecules B3 to XIAP leads to the ubiquitylation and degradation of XIAP. This results in stabilization of p53 and apoptosis (Fig. 6). These findings suggest that compounds that mimic the function of ARTS and specifically antagonize XIAP may have utility as cancer therapeutics by upregulating the levels of p53.

The ARTS-mimetic B3 increases the levels of p53 through its ability to bind the unique ARTS pocket in BIR3-XIAP. Similar to ARTS, B3 promotes the degradation of XIAP and increases the stability of p53 to promote apoptosis.

## Discussion

P53 is a tumor suppressor protein that acts as a major barrier against cancer development and progression. p53 functions primarily as a sequence-specific transcription factor [37]. A variety of studies over the years have identified positive and negative feedback loops in the p53 pathway [74]. Here we describe an amplification loop between p53 and ARTS (Fig. 6). Central to our model is the identification of XIAP as a novel E3 ligase of p53. In healthy cells, XIAP binds and degrades p53 through the UPS (Fig. 3C, D). Upon DNA damage, p53 binds to a distinct p53 binding element found within the ARTS promotor sequence and rapidly induces the transcription of ARTS (Supplementary Fig. 1) [52]. The upregulation of ARTS occurs in response to a wide variety of pro-apoptotic stimuli and is sufficient to induce apoptosis [15, 22]. ARTS acts mainly by antagonizing XIAP and reducing its levels by promoting its ubiquitylation and UPS-mediated degradation (Fig. 6). Here we show that ARTSXIAP-mediated ubiquitylation of p53, results in p53 stabilization and accumulation (Fig. 3D and Fig. 6). We consider two main models for the mechanism by which ARTS upregulates p53. A: upon binding of ARTS to XIAP, ARTS induces an allosteric conformational change in XIAP that causes auto-ubiquitylation and degradation of XIAP. As a consequence of XIAP self-conjugation and degradation, p53 levels increase. B: upon p53-mediated upregulation of ARTS, binding of ARTS to XIAP disrupts the interaction between XIAP and p53, which again reduces ubiquitylation and degradation of p53. Both models are not mutually exclusive, and a combination of both mechanisms may explain our results. Our finding that ARTS can be in a complex with p53 suggests the possibility that ARTS may form a ternary complex with XIAP and p53, which favors model A. In either case, the decrease in XIAP-mediated ubiquitylation of p53 causes its levels to rise and stimulate apoptosis.

We also describe a small-molecule ARTS-mimetic, B3, which was able to recapitulate key biochemical and functional properties of ARTS. First, B3 fits into the specific binding pocket of ARTS in the BIR3-XIAP. Second, B3, like ARTS, binds directly to XIAP (Fig. 4A). Third, B3 initiated the ubiquitylation and degradation of XIAP through the UPS (Fig. 4F). Fourth, B3 can promote the upregulation of p53 by downregulating XIAP levels to trigger apoptosis. Collectively, these results indicate that B3 is a small-molecule ARTS-mimetic. Moreover, they suggest that the ARTS binding pocket in BIR3-XIAP can be targeted to increase p53 levels, and this may provide a new approach for developing p53-based anti-cancer therapeutics.

The pursuit of p53-targeted therapy began with the identification of compounds capable of restoring wild-type p53 functions or eliminating mutant p53 [75, 76]. The reactivation of p53 in cancers containing low levels of WT-p53 through inhibition of MDM2 has been challenging, since it causes widespread cytotoxicity due to activation of WT p53 in normal tissues [37, 65, 68]. In addition, monotherapy with MDM2 antagonists is insufficient to suppress tumor progression [37, 65, 68]. Therefore, many subsequent efforts have focused on identifying promising drug combinations [37, 65]. Our results show that B3 can kill a wide range of cancer cell types but leaves normal PBMCs intact (Supplementary Fig. 2).

p53 plays a major role in promoting apoptosis in response to chemotherapy-induced DNA damage [77]. Many cancers can escape apoptosis by overexpressing XIAP [–20, –34, –56, 78–84]. Therefore, XIAP has become an attractive target for the development of anti-cancer drugs [20, 32, 56, 85]. Most efforts to target IAPs have focused on developing IBM (IAP Binding Motif) mimetics [86–92]. However, most of these compounds bind and degrade primarily cIAPs, that can also result in hyper-inflammation and widespread toxicity [91, 93–96]. Therefore, there is a strong interest in developing specific inhibitors of XIAP. The identification of the ARTS-mimetics B3 shows that it is possible to target the interaction between XIAP and p53 with specific small-molecule ARTS-mimetic compounds. Therefore, our results provide the foundation for developing a new class of small-molecules that target the unique binding site of ARTS within XIAP, and can be effective against a wide range of cancers.

### Supplementary Information

Below is the link to the electronic supplementary material.

Supplementary file1 (DOCX 6713 KB)

## Data Availability

Not applicable.
