# Supplementary material for: ARTS and small-molecule ARTS mimetics upregulate p53 levels by promoting the degradation of XIAP
Source: Apoptosis. 2024 Apr 29;29(7-8):1145–60. doi: 10.1007/s10495-024-01957-2 (PMC11263447; doi:10.1007/s10495-024-01957-2)

# Supplementary figures

**Supplementary figure.1** Real-Time PCR results. WT HCT116 and p53 KO HCT116 cells were induced for apoptosis with UV for the indicated time points. Real-Time PCR was performed, and cDNAs were normalized to GPI (housekeeping gene). Under apoptotic conditions, a strong upregulation of ARTS was observed after 5 minutes of UV treatment in WT cells. The graph represents three independent experiments and is plotted as mean ± SEM. Statistical analysis was carried out by GraphPad Prism software using a two-way ANOVA. Statistical significance is denoted by *, **, or *** to indicate P<0.05, P<0.001, or P<0.0001, respectively.

**Supplementary figure.2** Differential cancer cell killing by B3. IC50 values (µM) for a panel of 94 tumor cell lines (NCI-60 extended panel) treated with B3. Six concentrations of B3 were used to calculate the IC50 values for each cell line. Differential cell death effects of B3 on various cancer cell lines were observed. Normal PBMC (peripheral blood mononuclear cells) were resistant to killing by B3.

**Supplementary figure.3** Flow cytometry plots of BiFC assays show the percentage of cells expressing fluorescent YFP (Venus, P1) and PE (red, P2) signals, indicating close proximity between each pair of proteins. FACS analysis results reveal the formation of a complex between p53 and ARTS upon treatment with Etoposide.

**Supplementary figure.4** Flow cytometry plots of BiFC assays show the percentage of cells expressing fluorescent YFP (Venus, Q1) and PE (red, Q4) signals, indicating close proximity between each pair of proteins. FACS analysis show that B3 disrupts the complex between XIAP and p53.


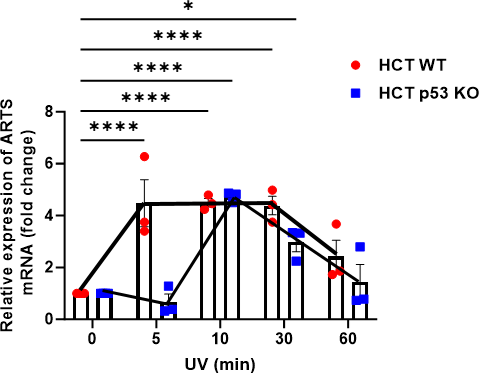


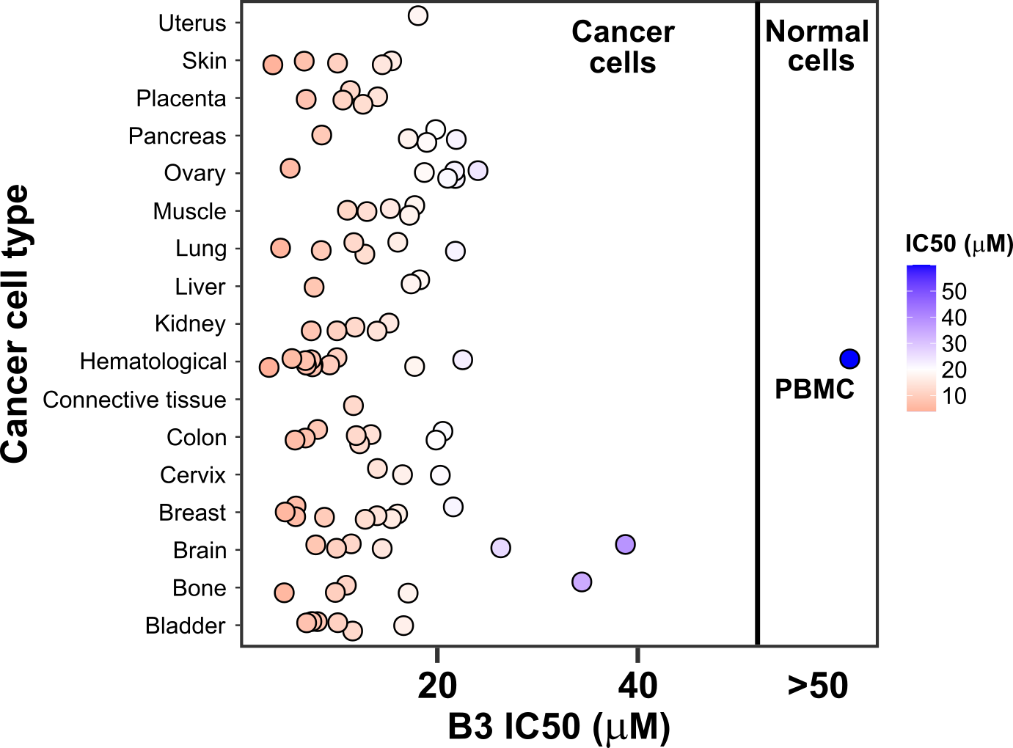


**Repeat.1**


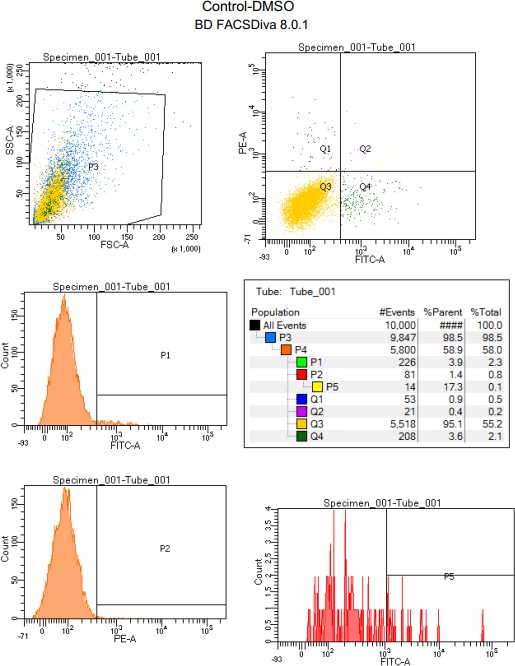


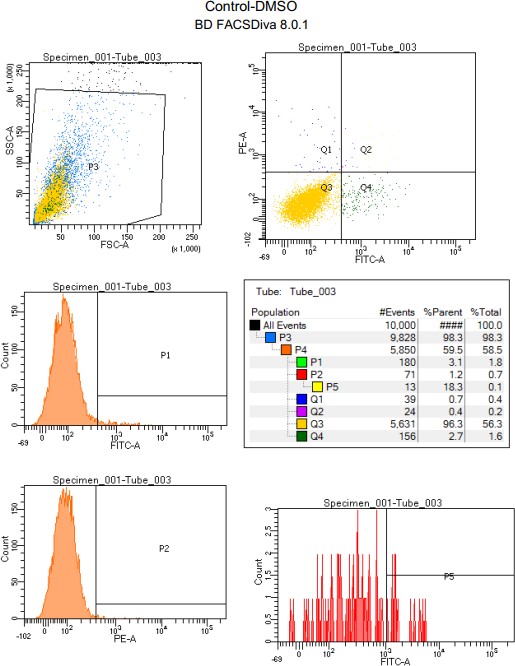
**Repeat.1**


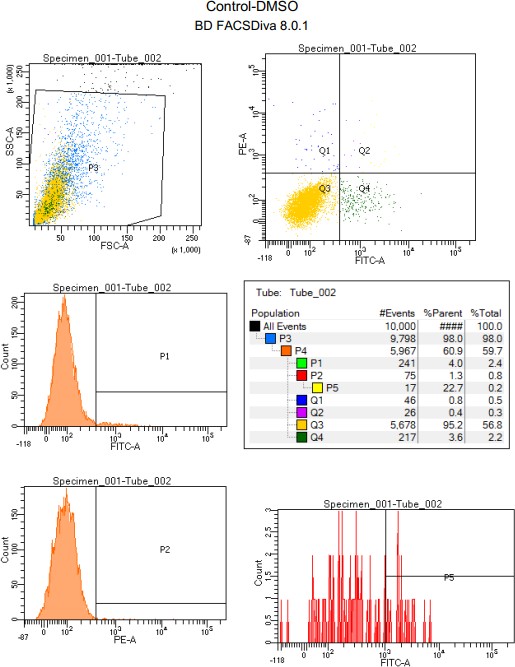
**Repeat.1**


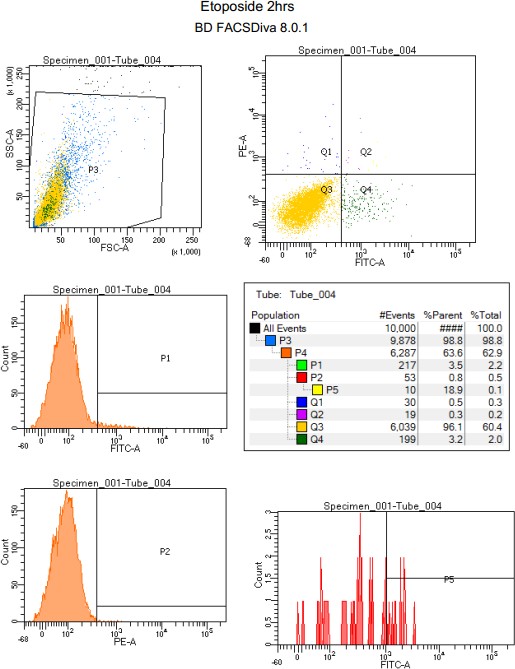
**Repeat.1**


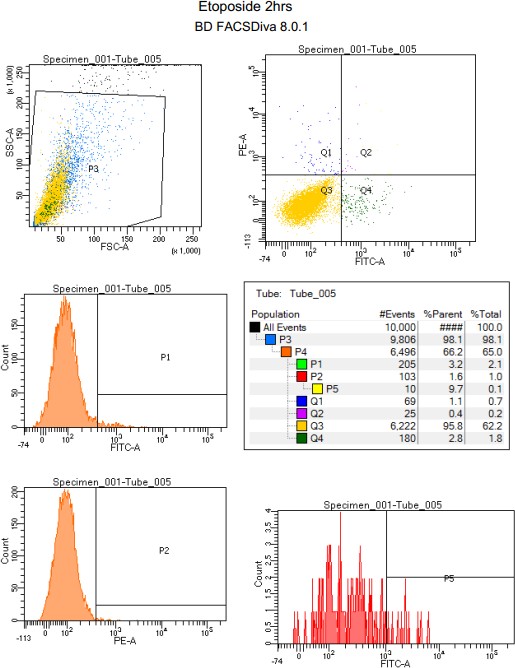
**Repeat.1**


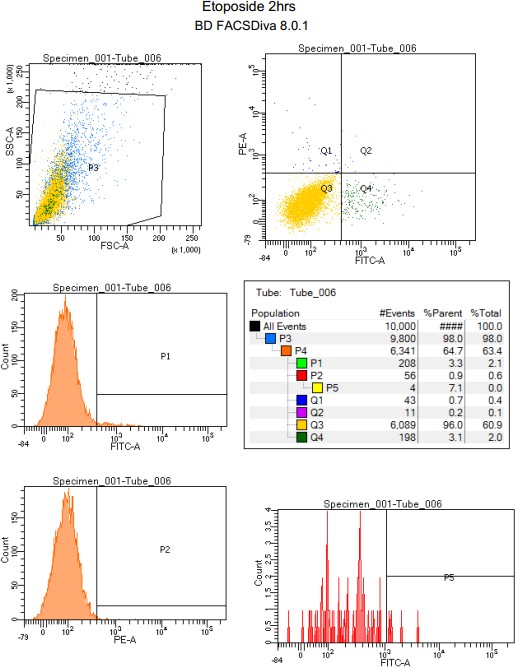
**Repeat.1**


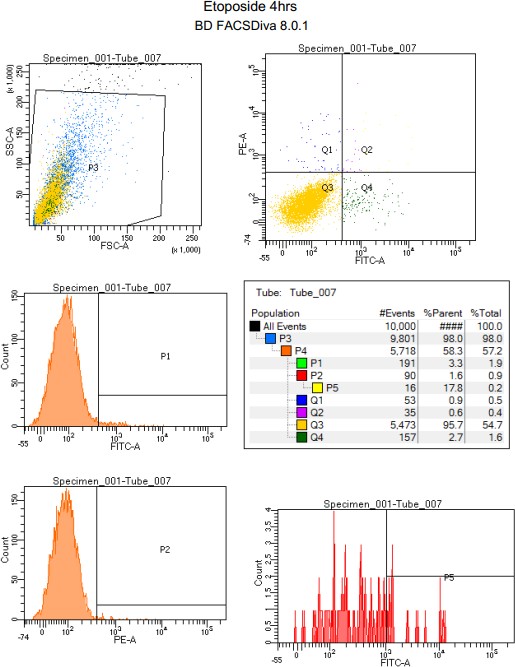
**Repeat.1**

**Repeat.1**


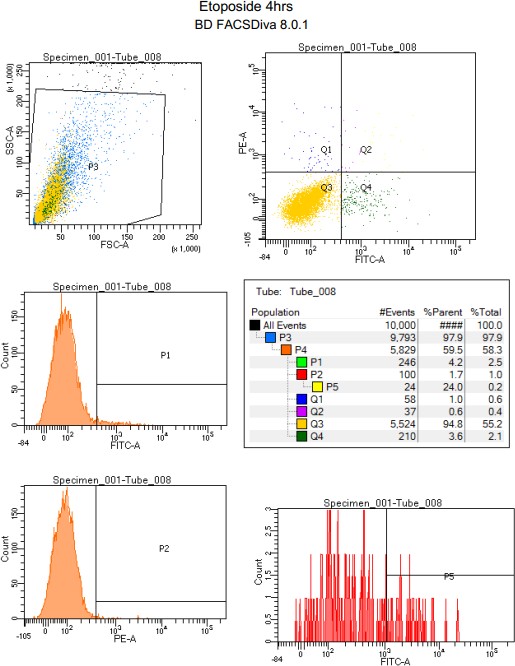


**Repeat.1**


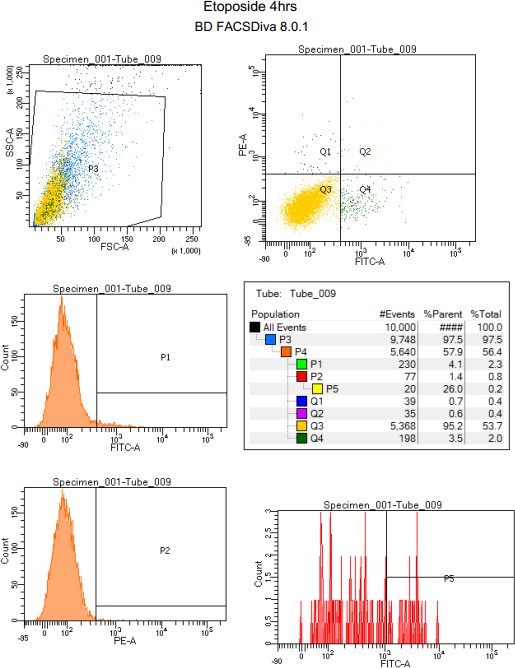


**Repeat.1**


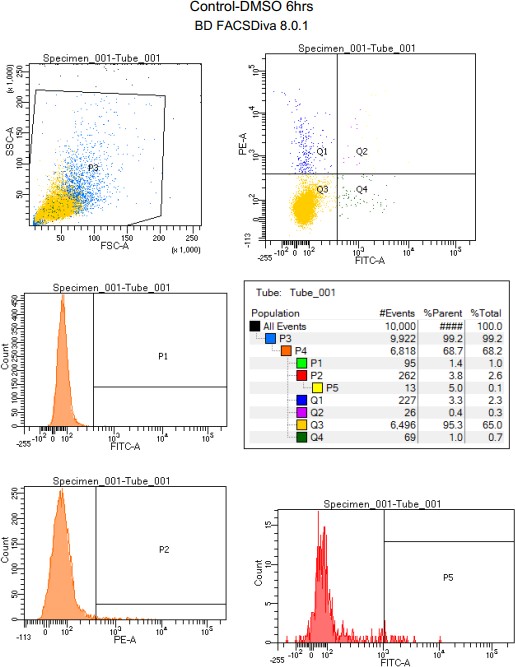


**Repeat.1**


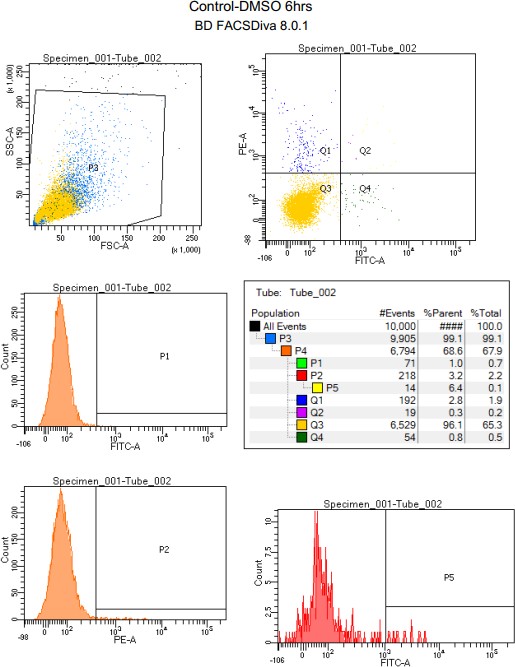


**Repeat.1**


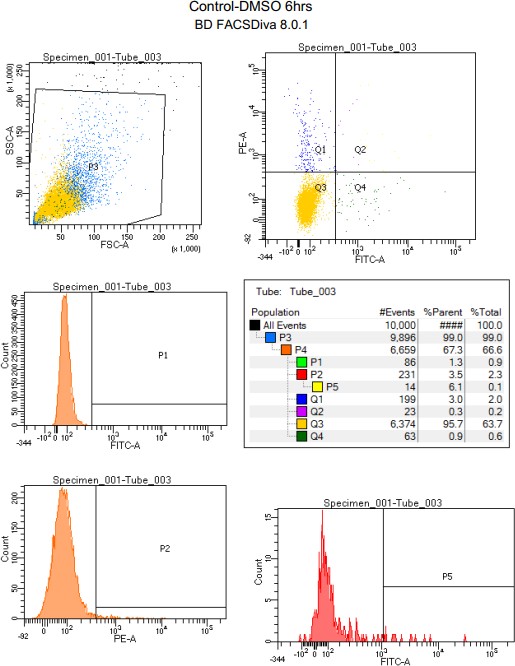


**Repeat.1**


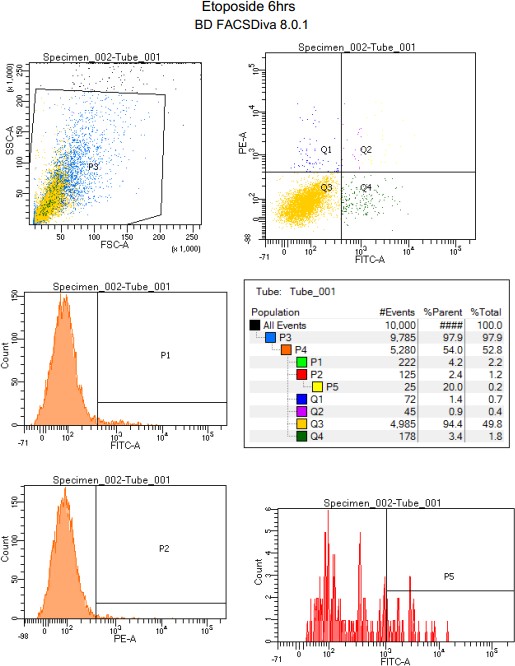


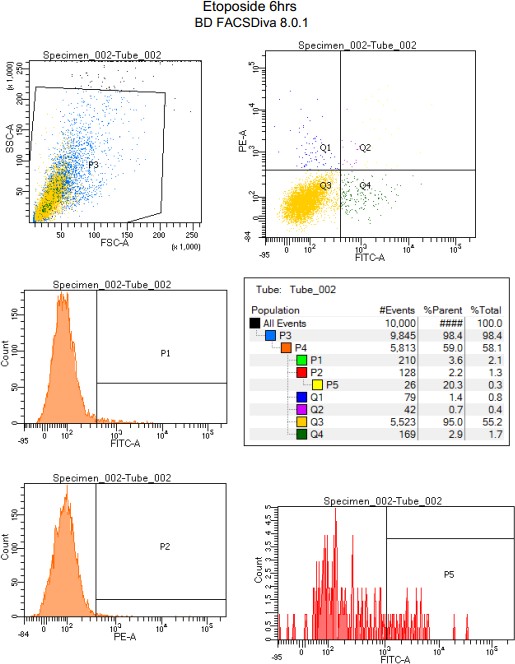
**Repeat.1**

**Repeat.1**


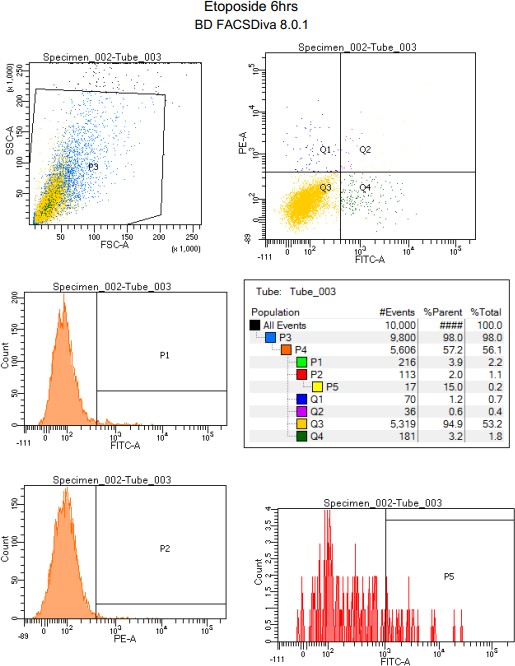


**Repeat.1**


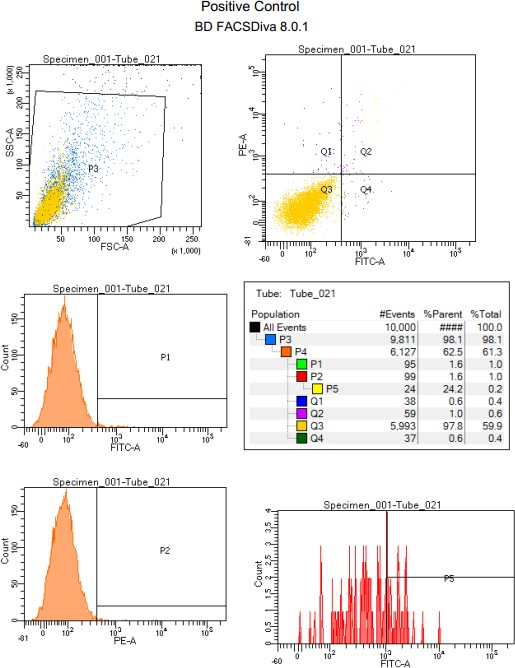


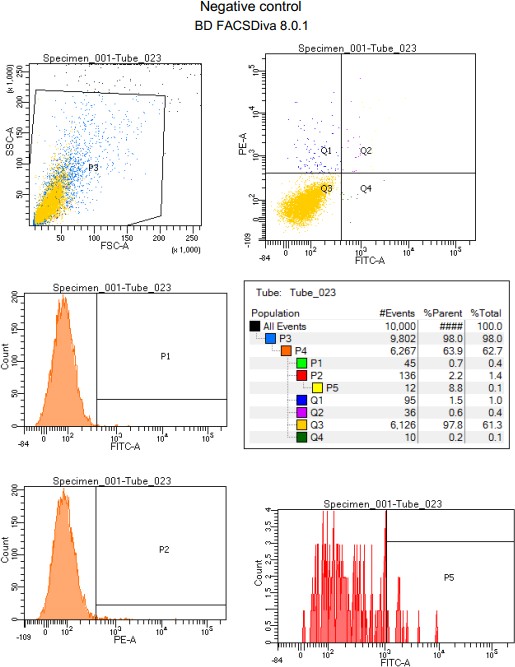
**Repeat.1**


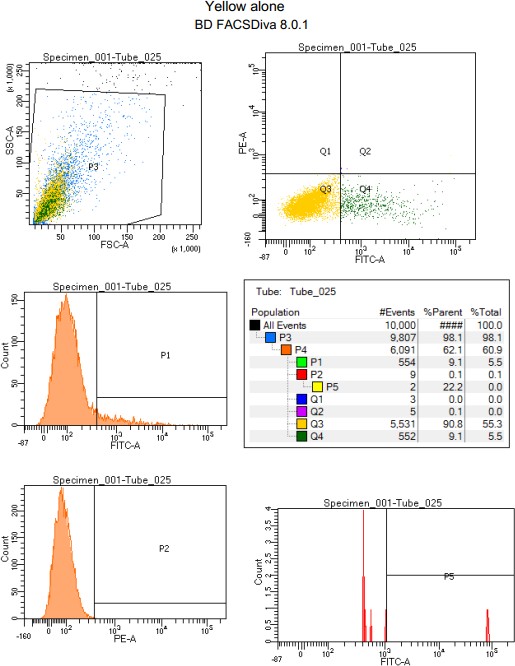
**Repeat.1**


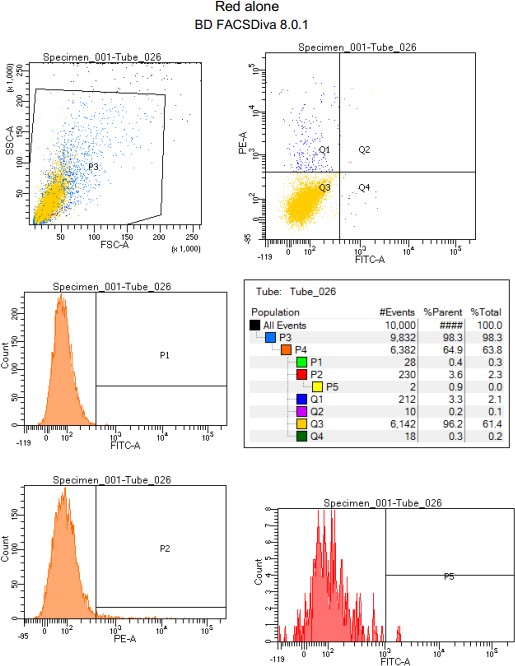
**Repeat.1**

**Repeat.2**


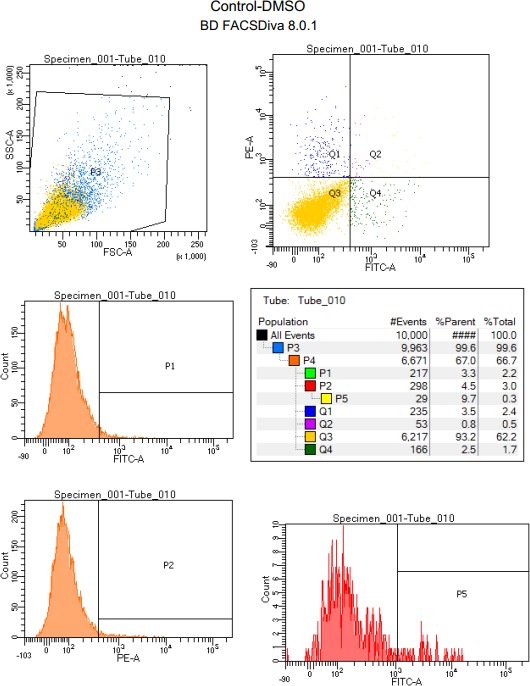


**Repeat.2**


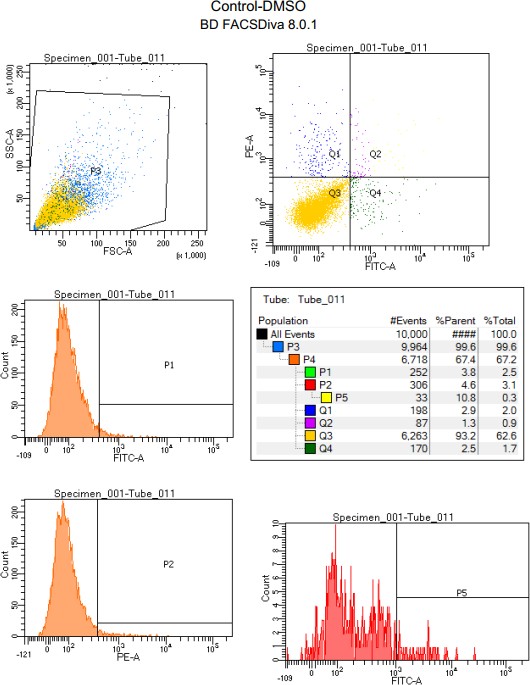


**Repeat.2**


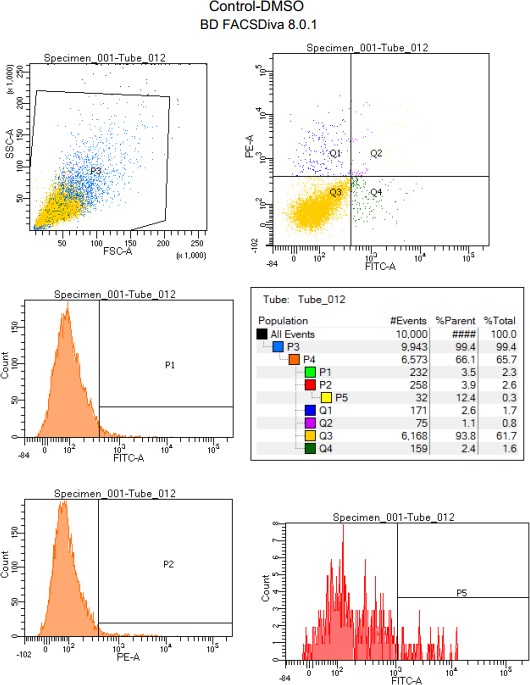


**Repeat.2**


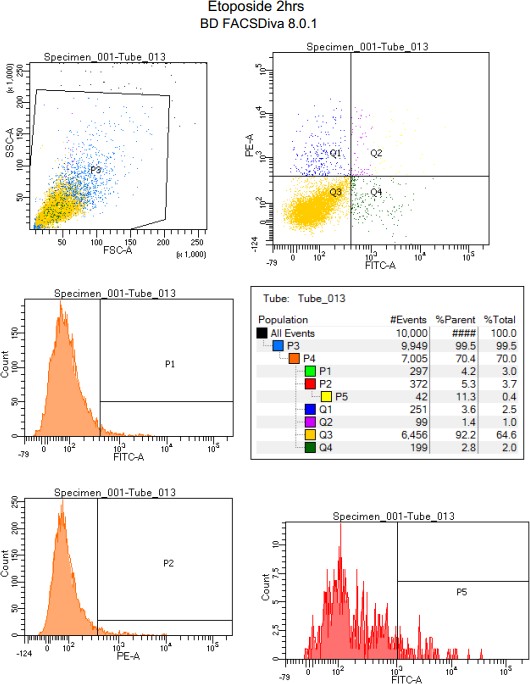


**Repeat.2**


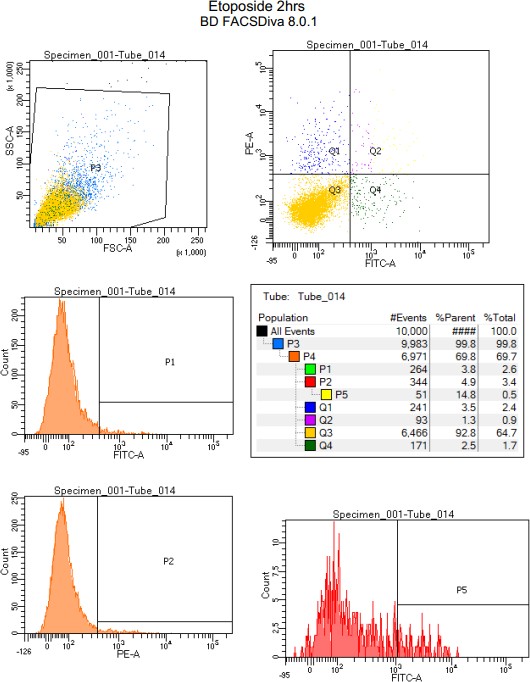


**Repeat.2**


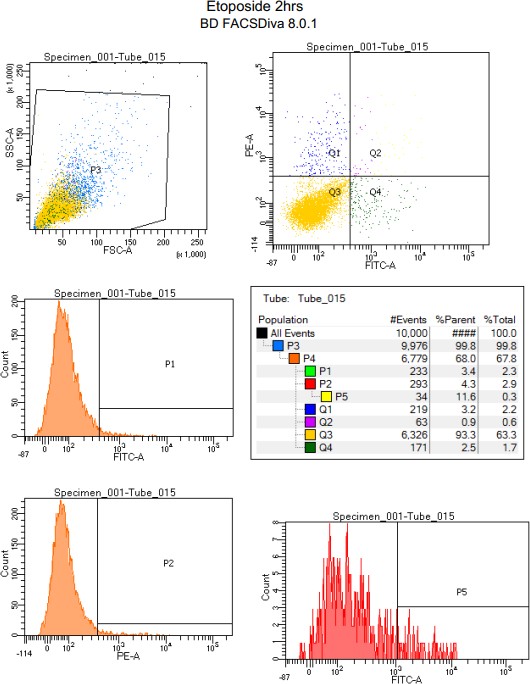


**Repeat.2**


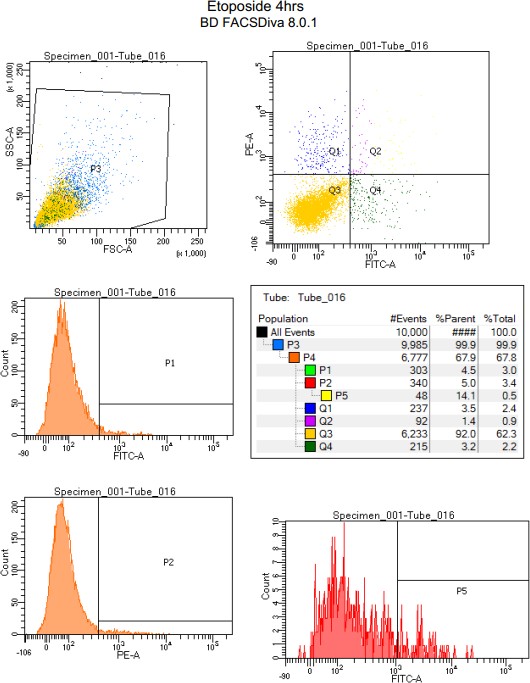


**Repeat.2**


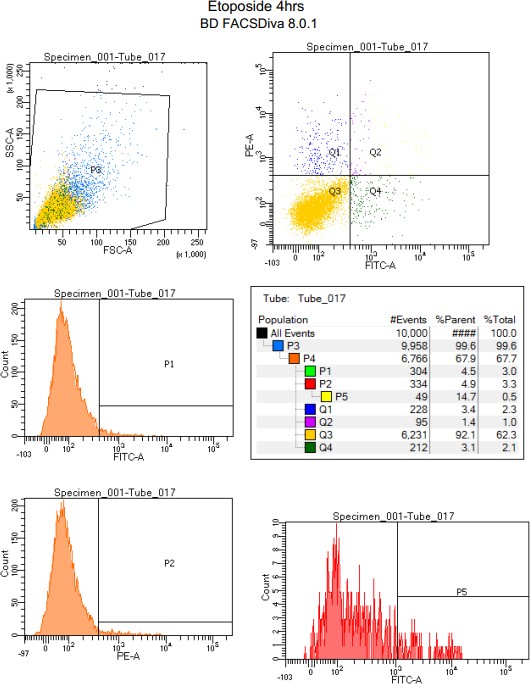


**Repeat.2**


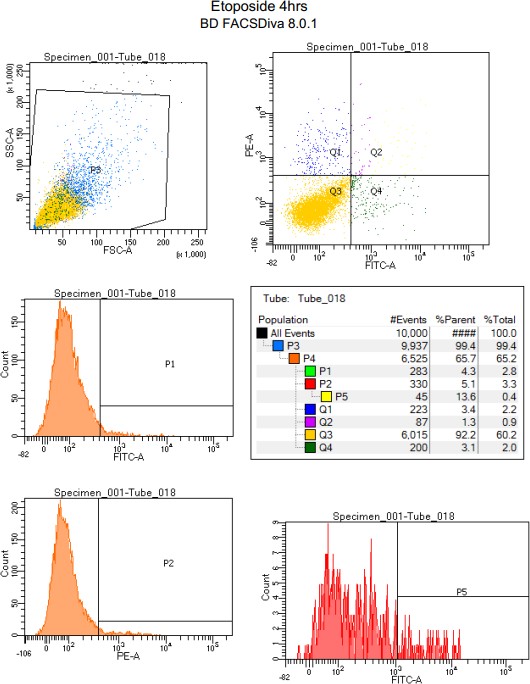


**Repeat.2**


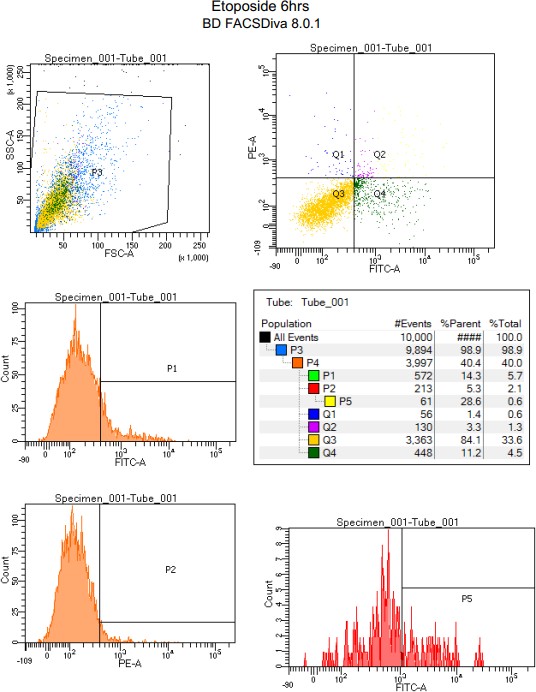


**Repeat.2**


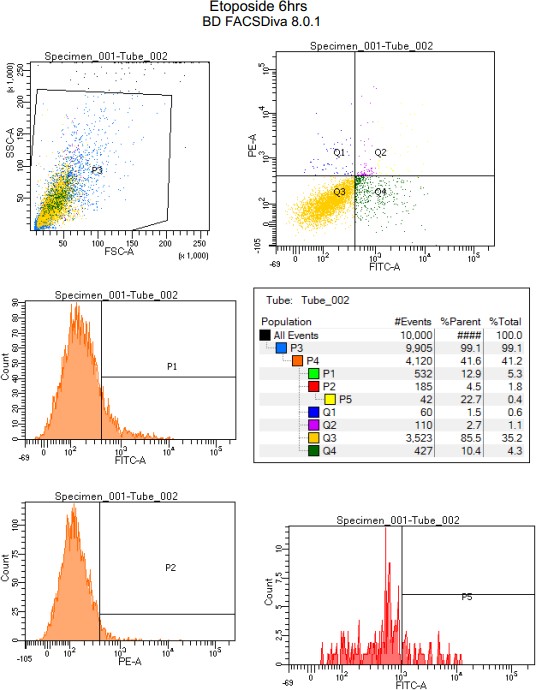


**Repeat.2**


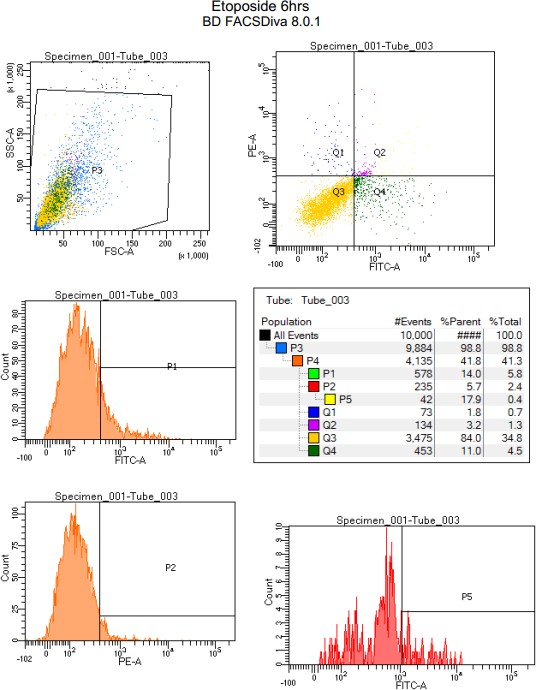


**Repeat.2**


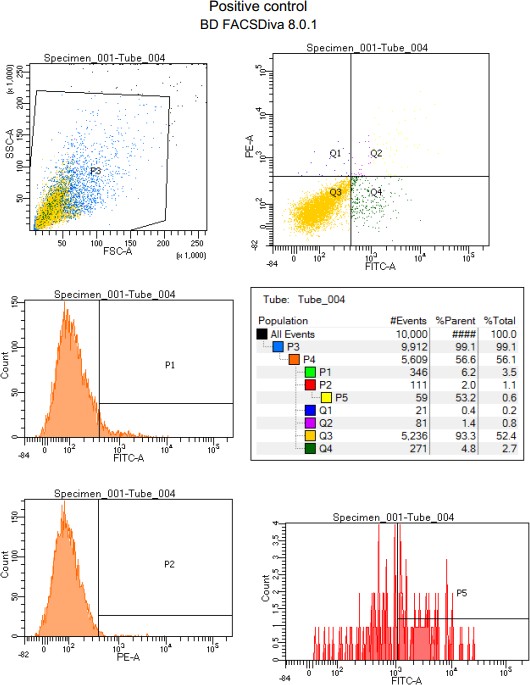


**Repeat.2**


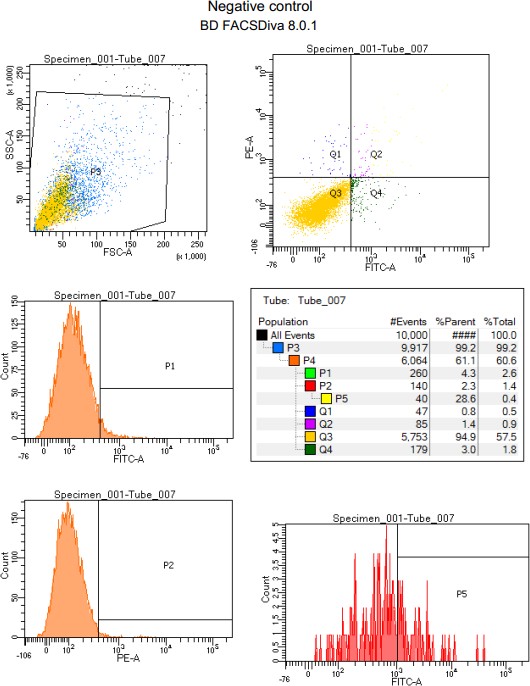


**Repeat.2**


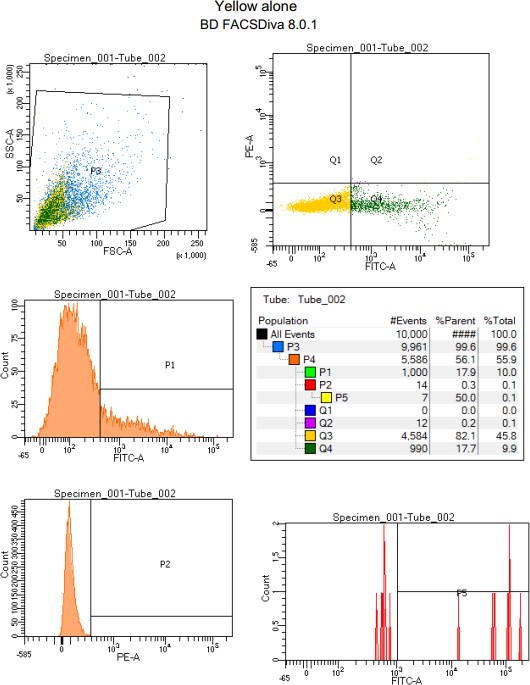


**Repeat.2**


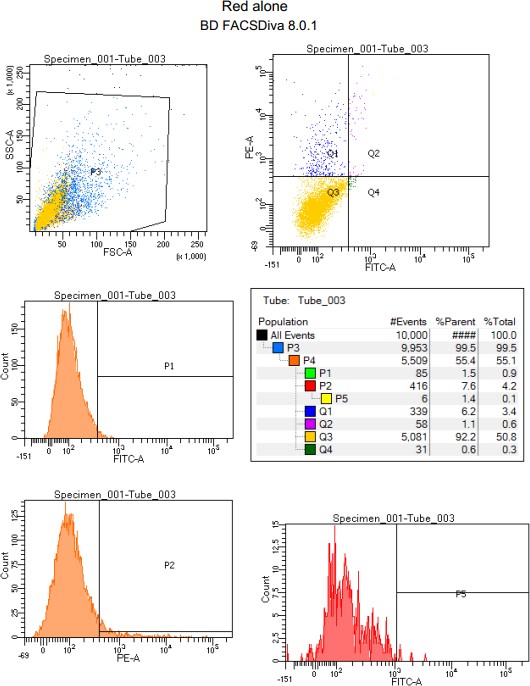


**Repeat.3**


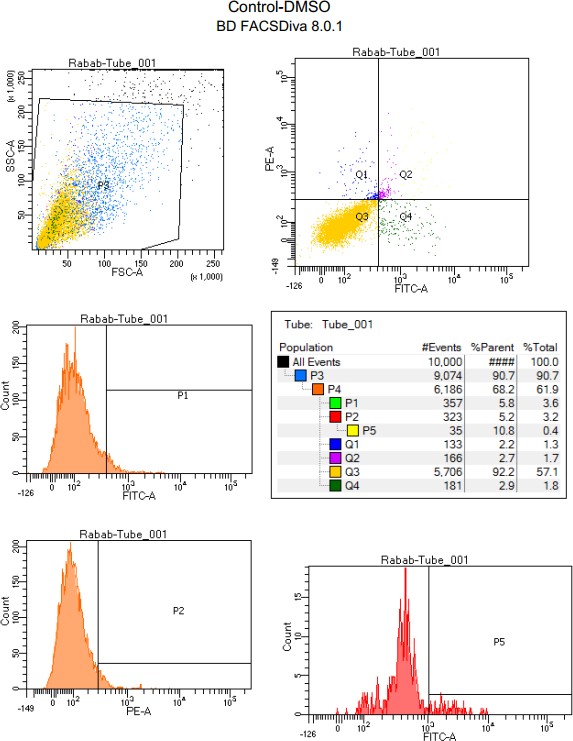


**Repeat.3**


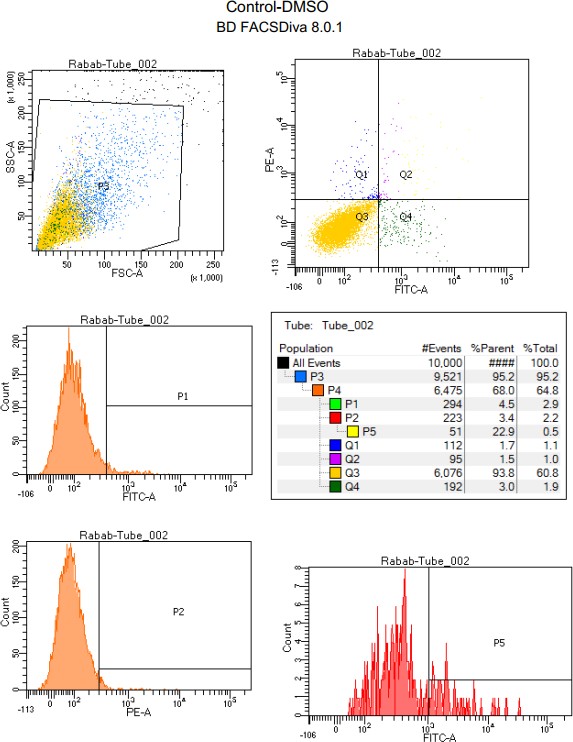


**Repeat.3**


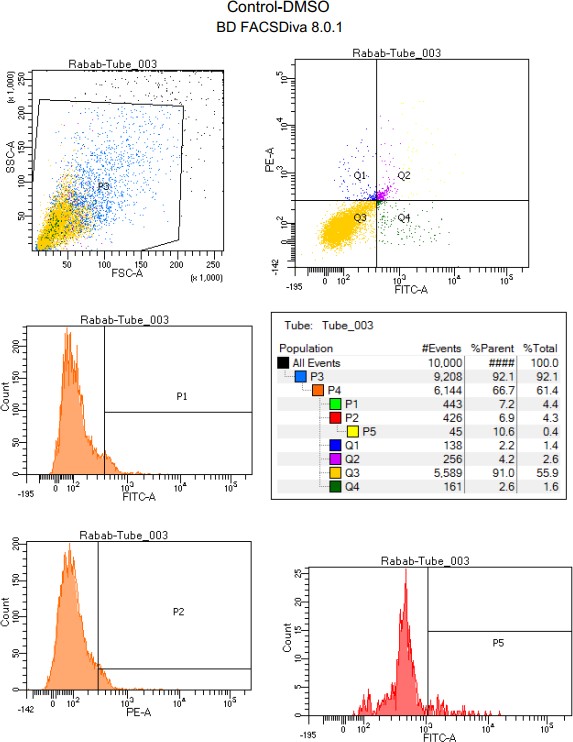


**Repeat.3**


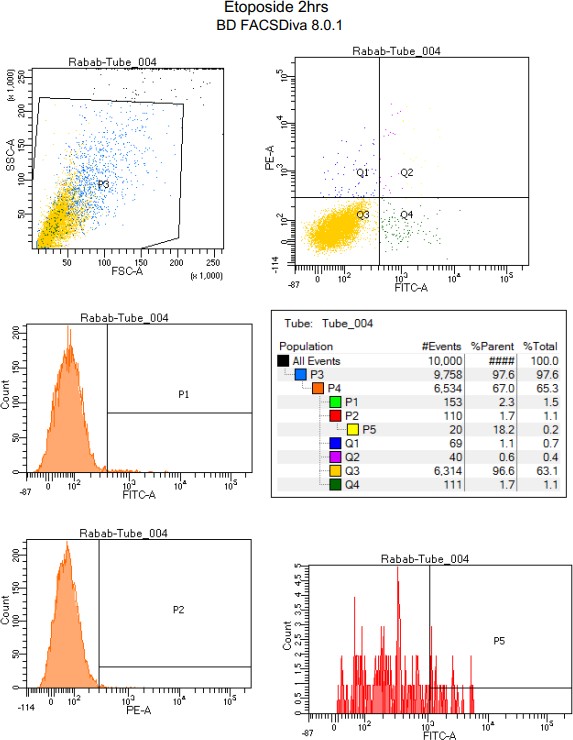


**Repeat.3**


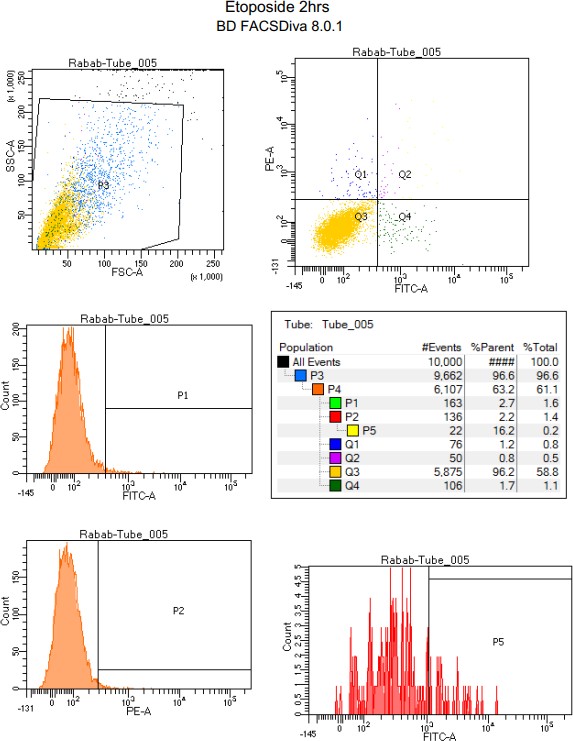


**Repeat.3**


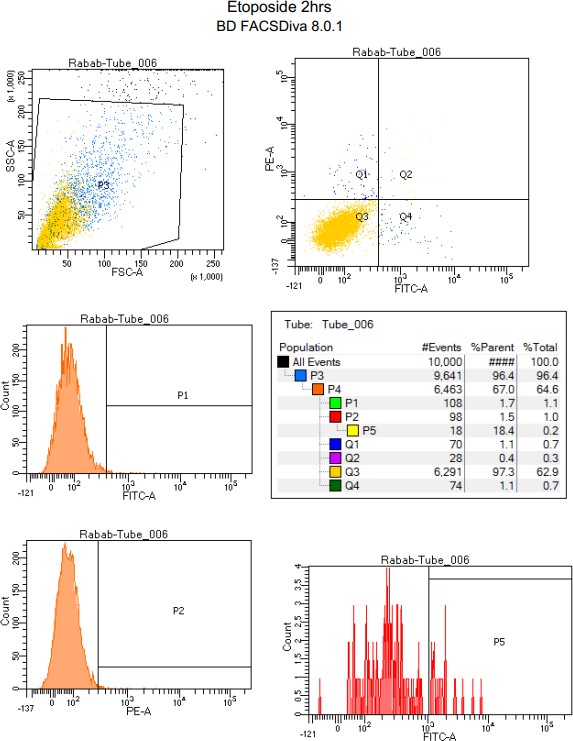


**Repeat.3**


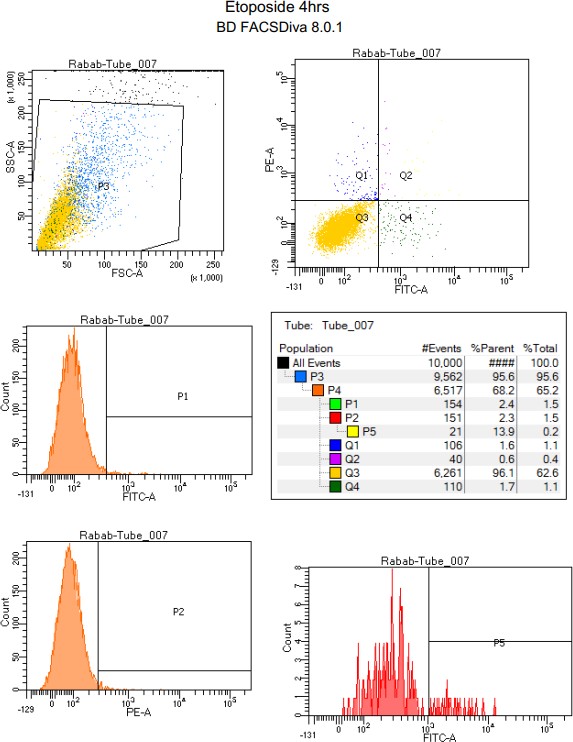


**Repeat.3**


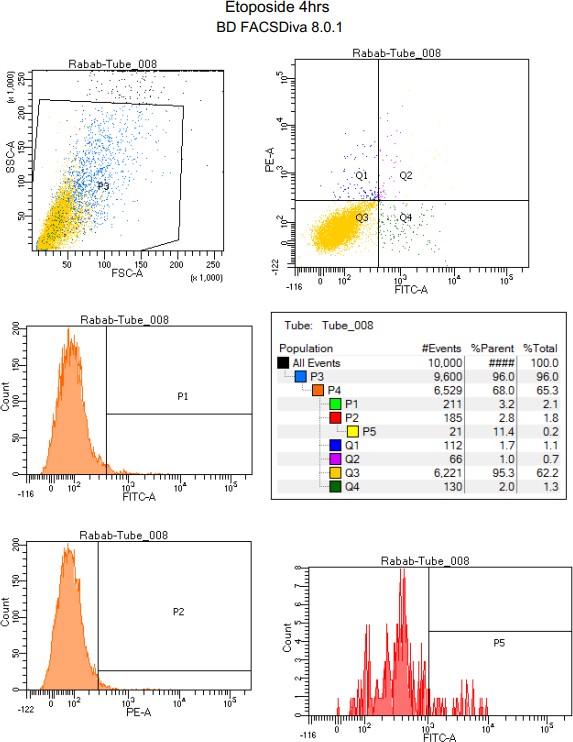


**Repeat.3**


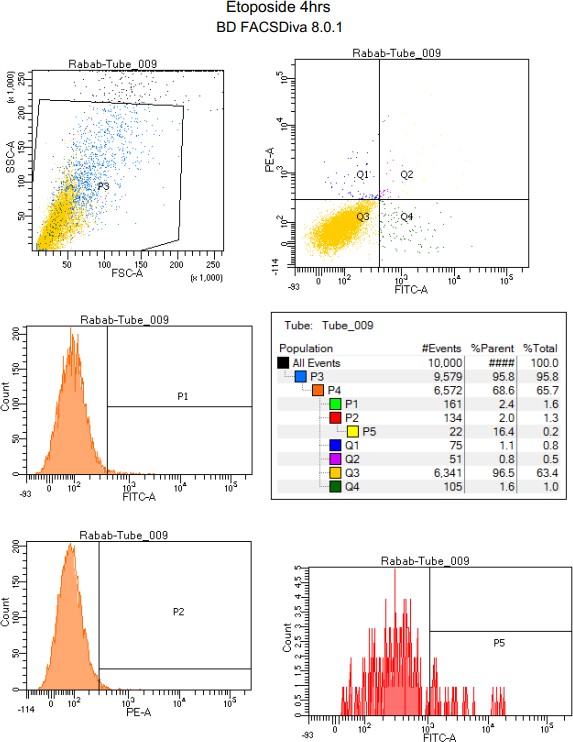


**Repeat.3**


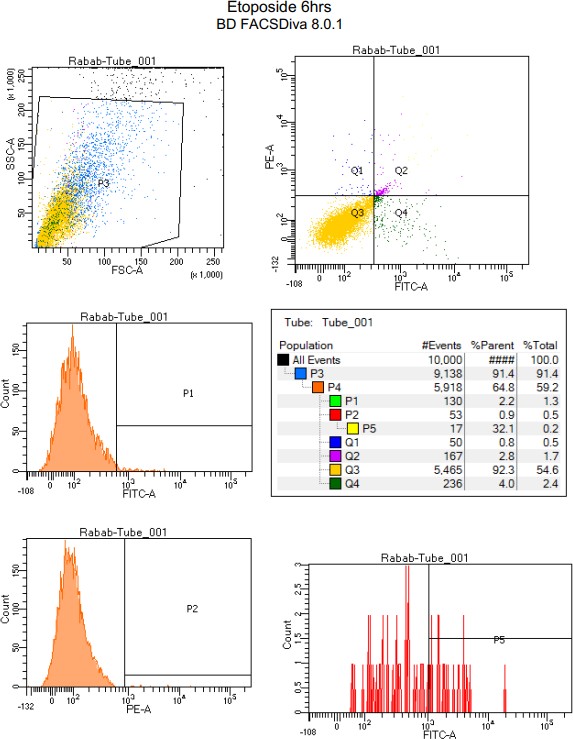


**Repeat.3**


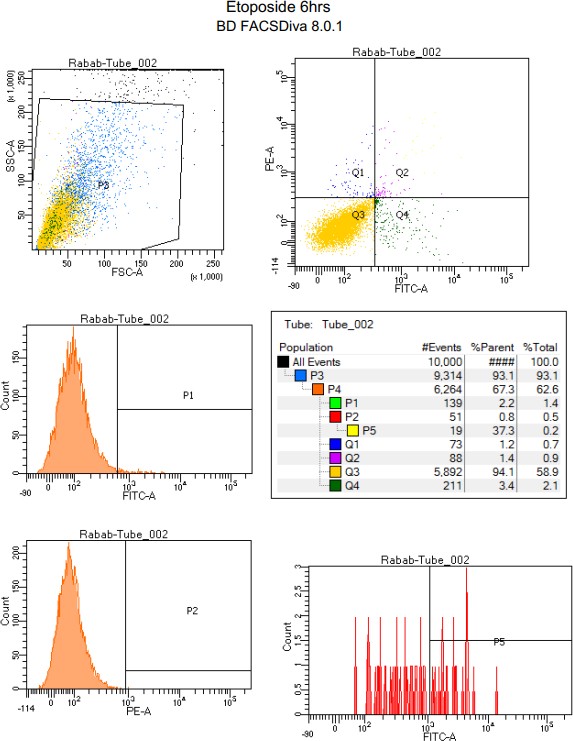


**Repeat.3**


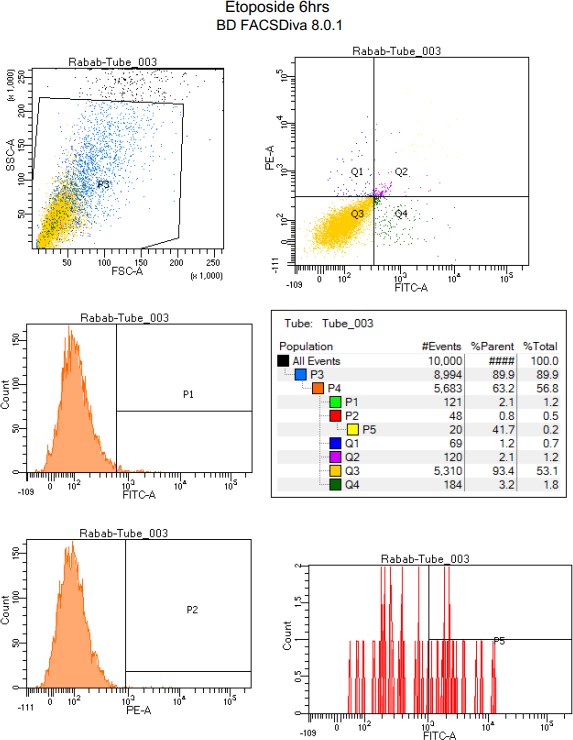


**Repeat.3**


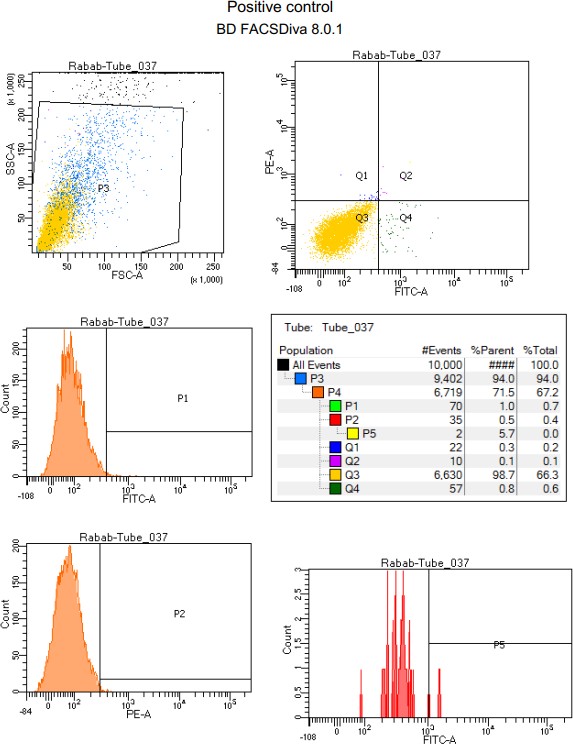


**Repeat.3**


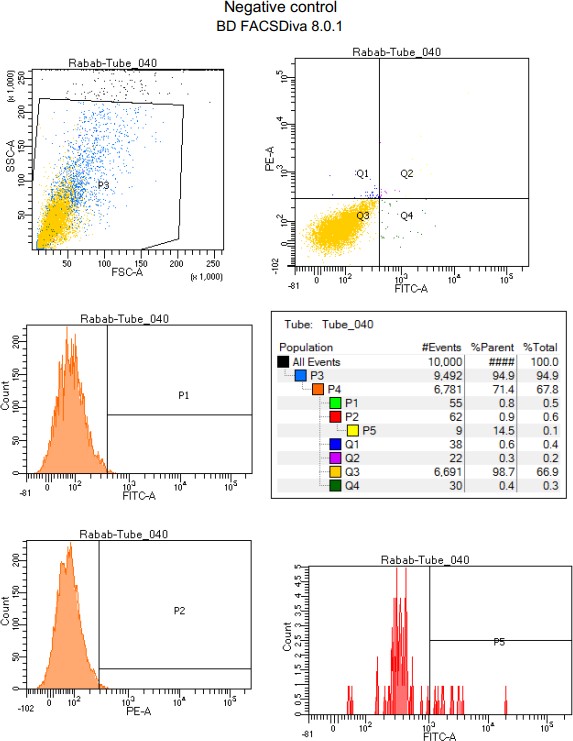


**Repeat.3**


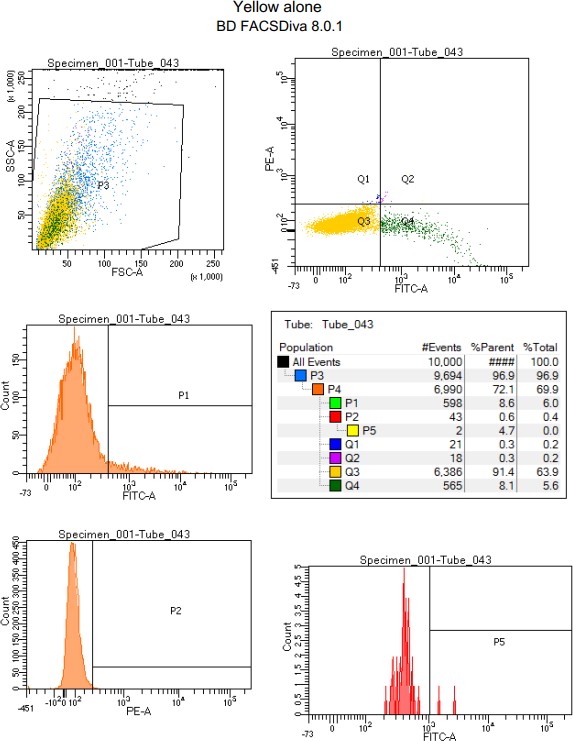


**Repeat.3**


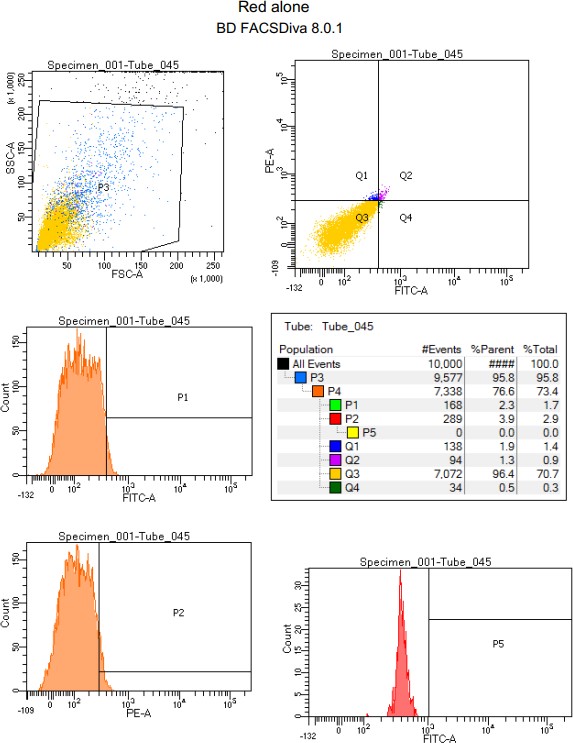


**Repeat.1**


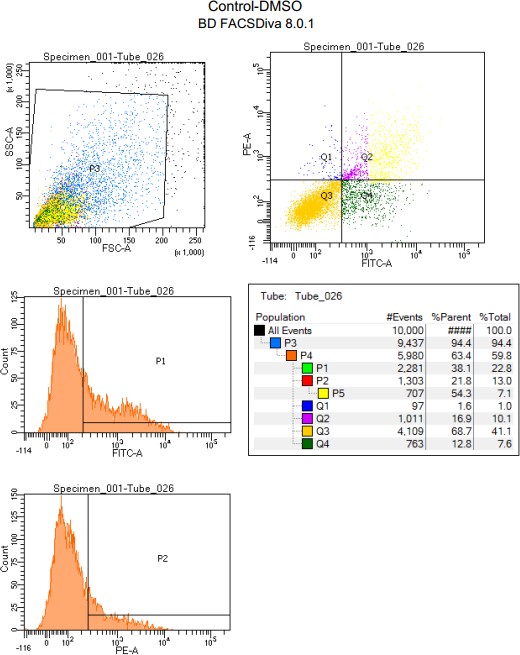


**Repeat.1**


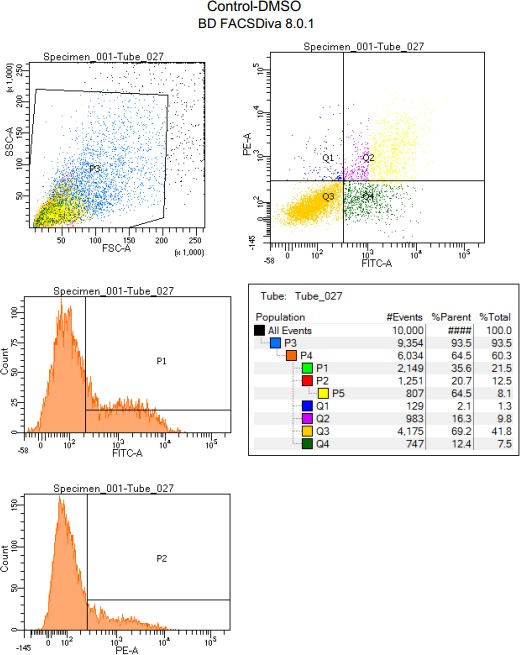


**Repeat.1**


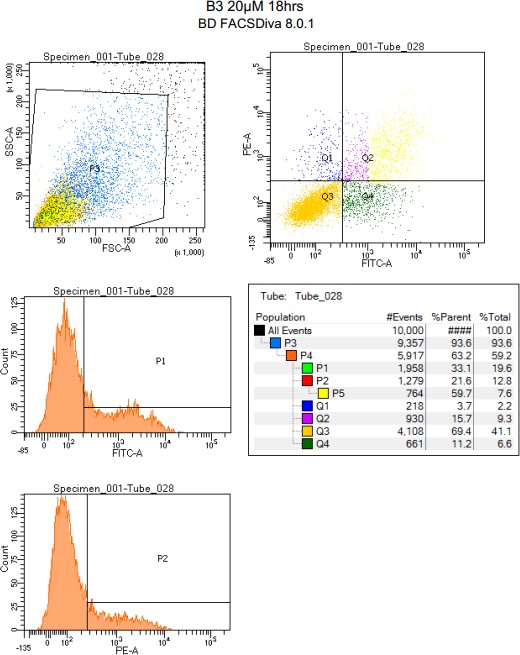


**Repeat.1**


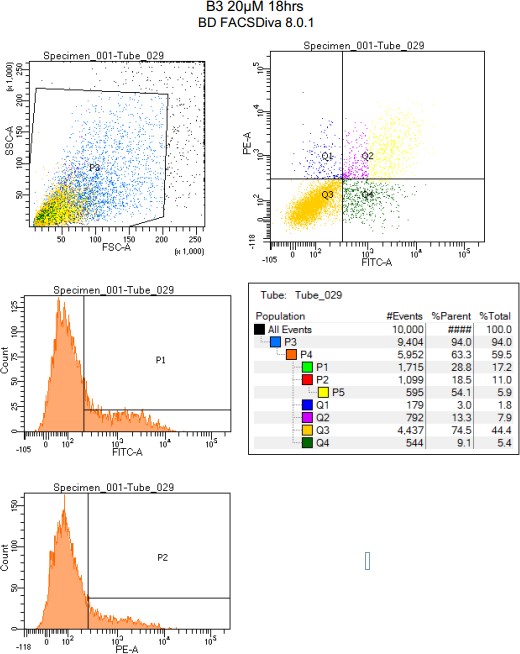


**Repeat.1**


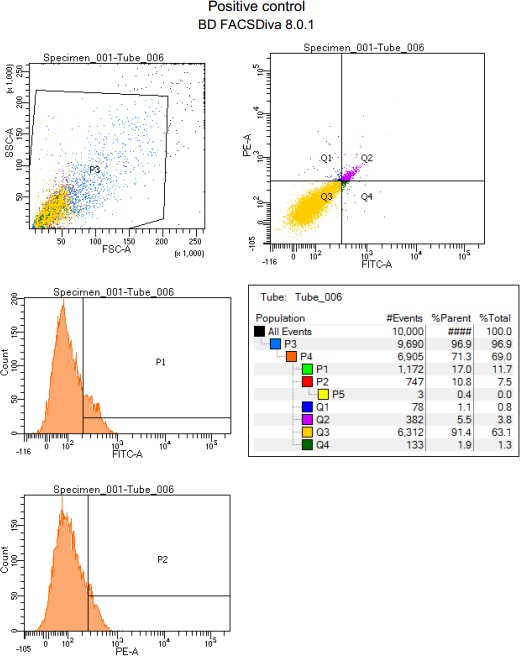


**Repeat.1**


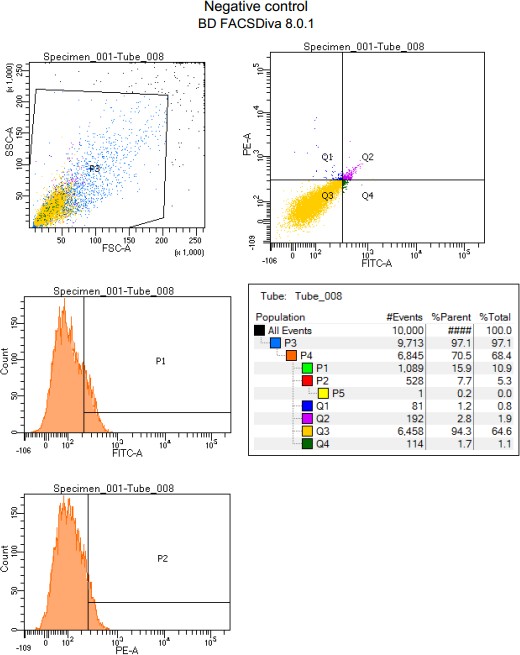


**Repeat.1**


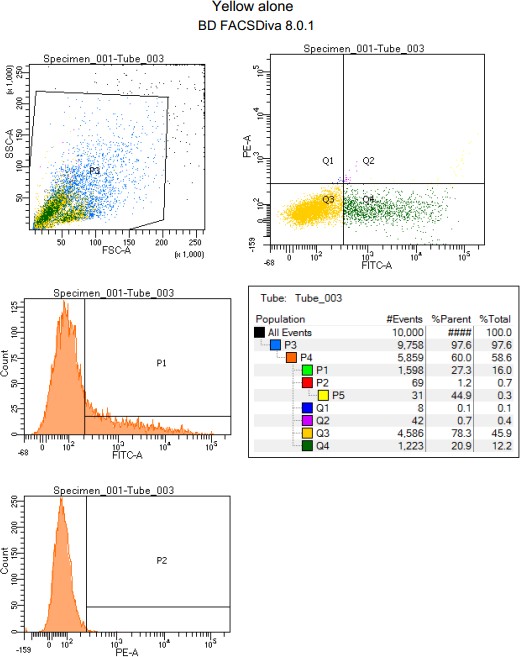


**Repeat.1**


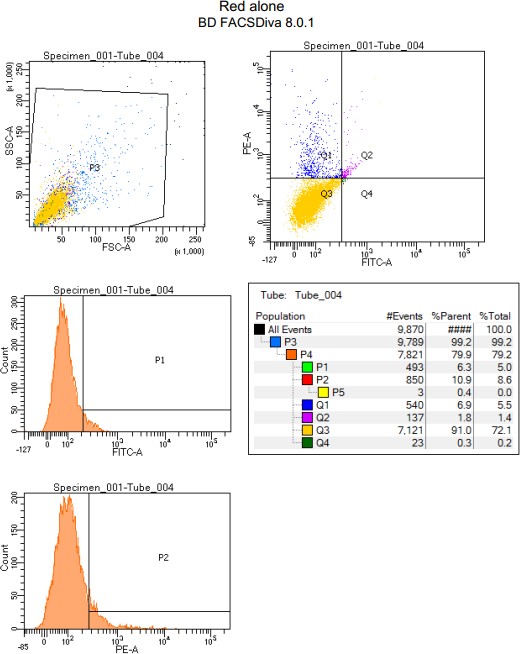


**Repeat.2**


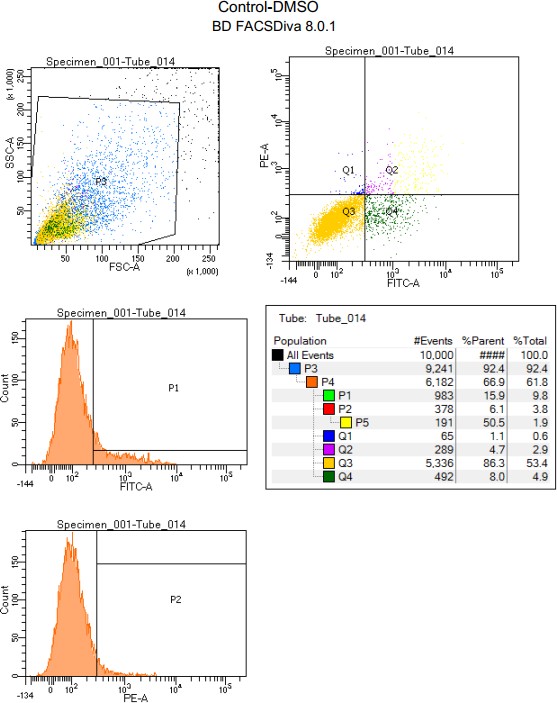


**Repeat.2**


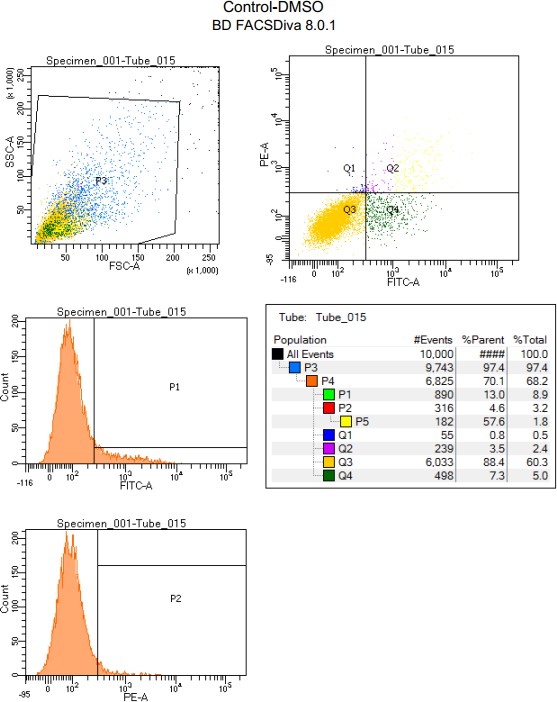


**Repeat.2**


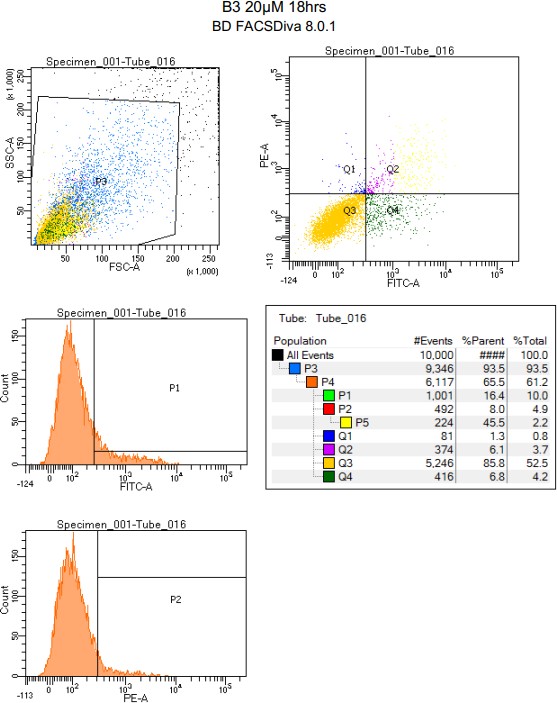


**Repeat.2**


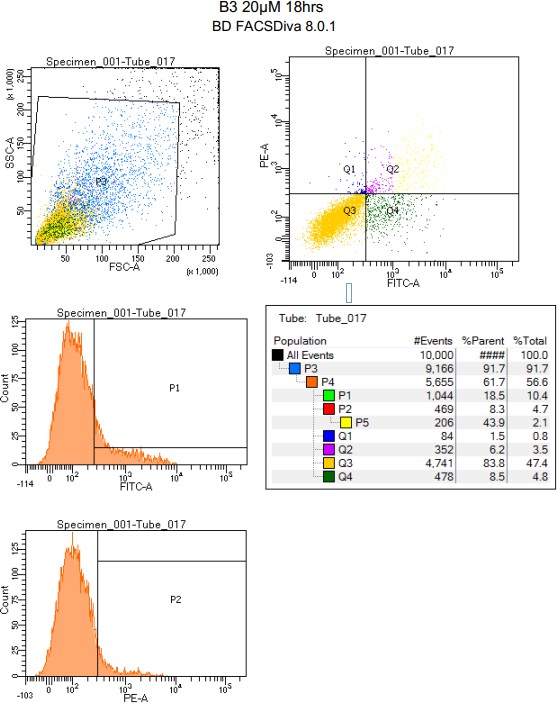


**Repeat.2**


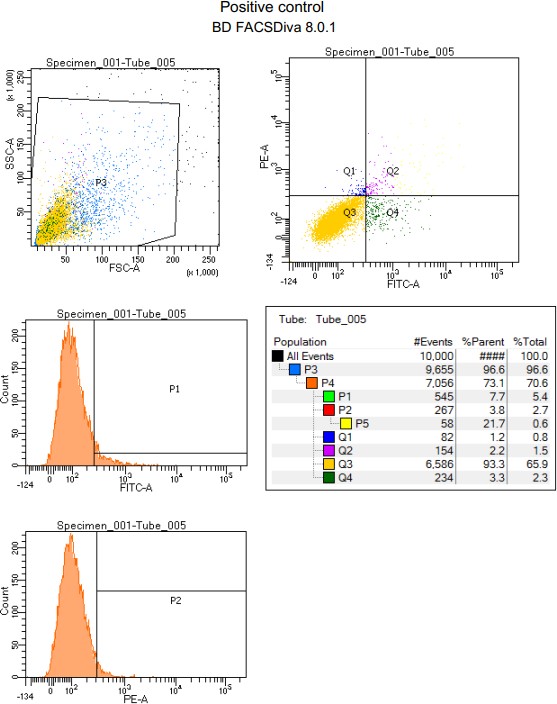


**Repeat.2**


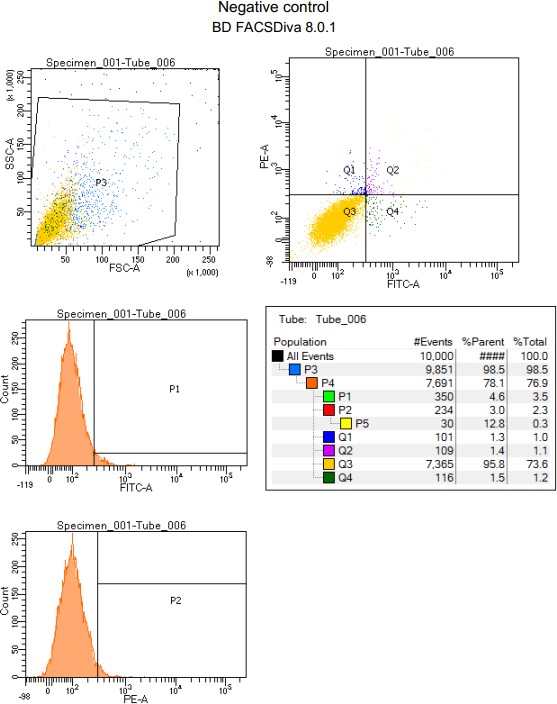


**Repeat.2**


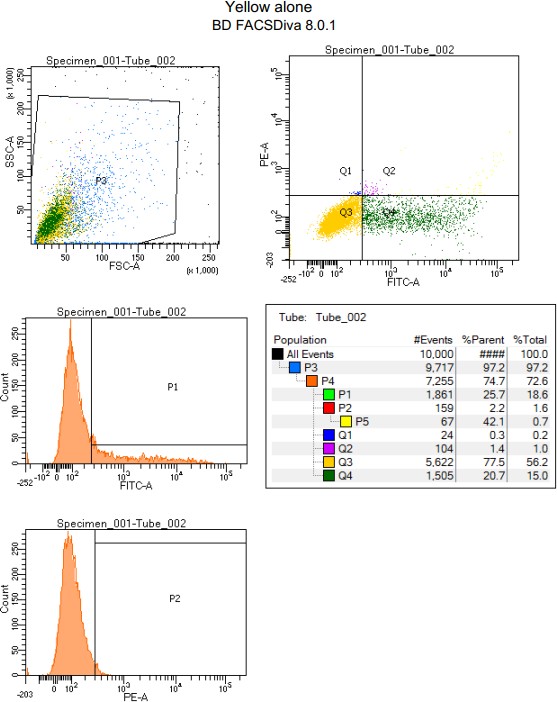


**Repeat.2**


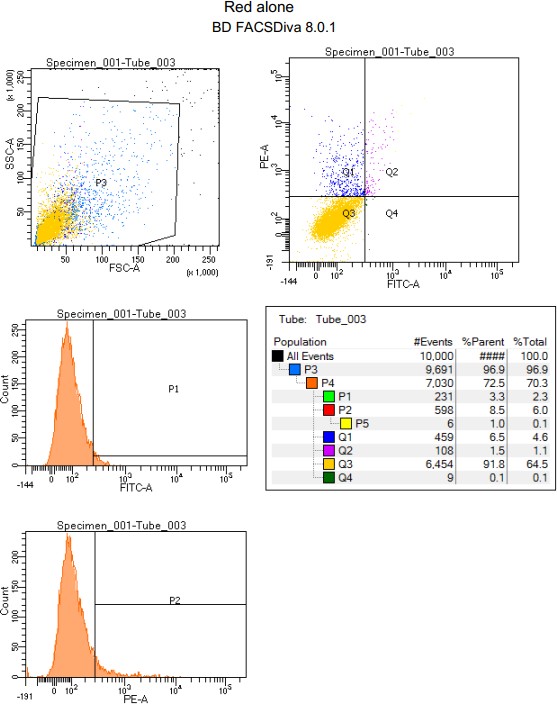


**Repeat.3**


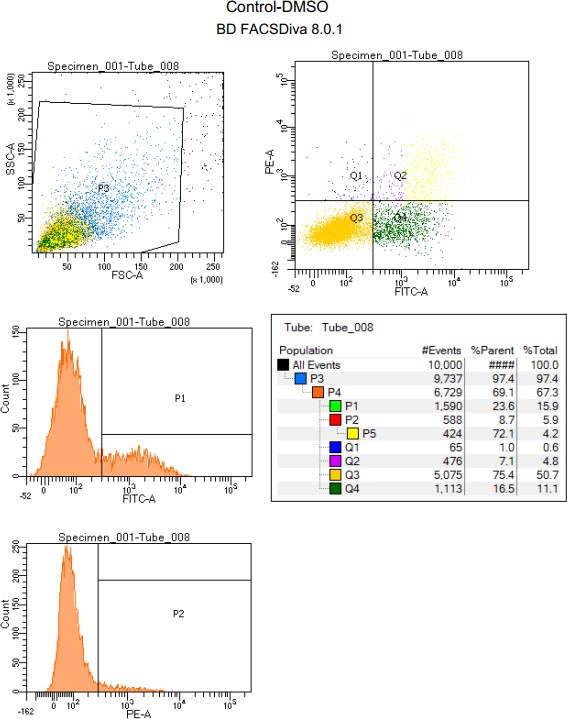


**Repeat.3**


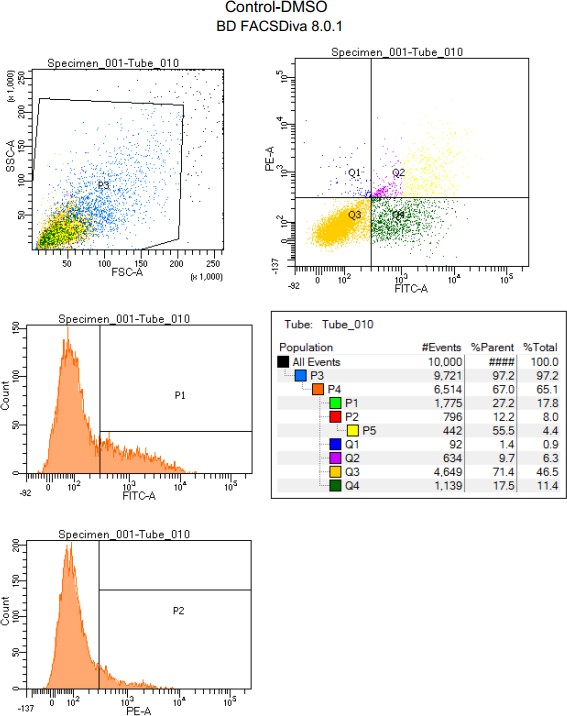


**Repeat.3**


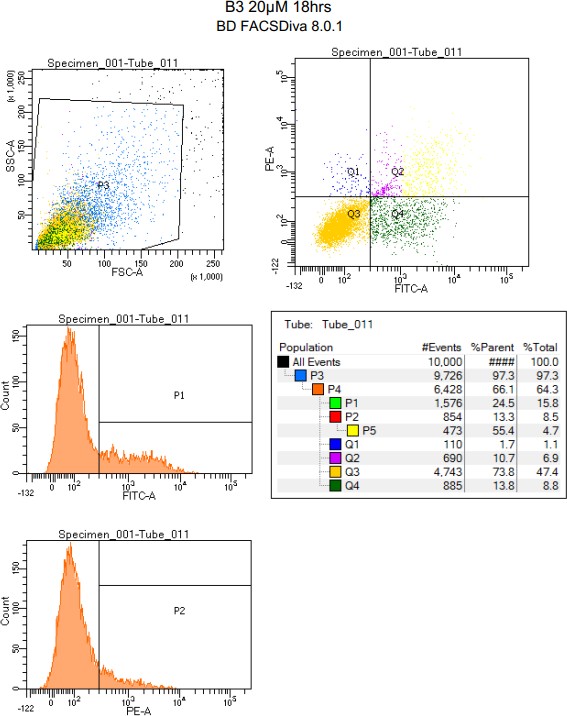


**Repeat.3**


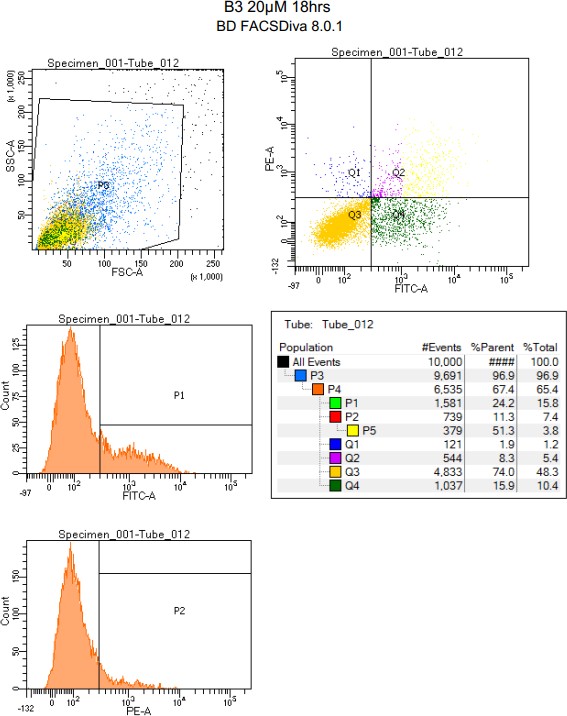


**Repeat.3**


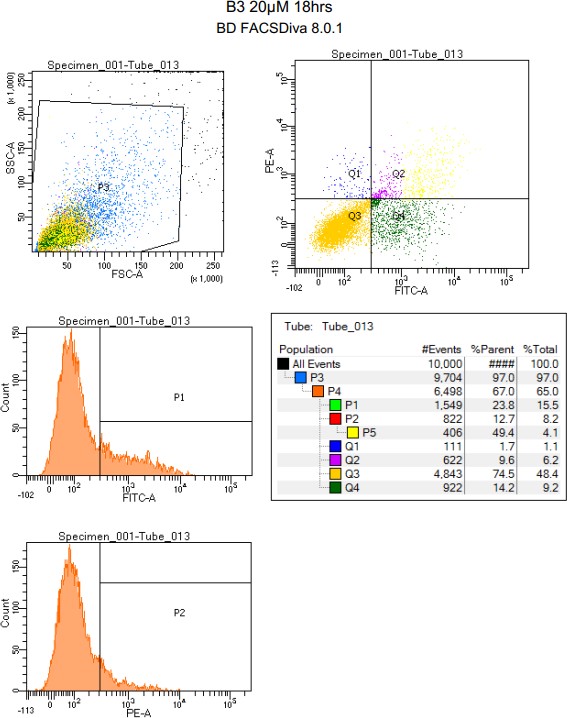


**Repeat.3**


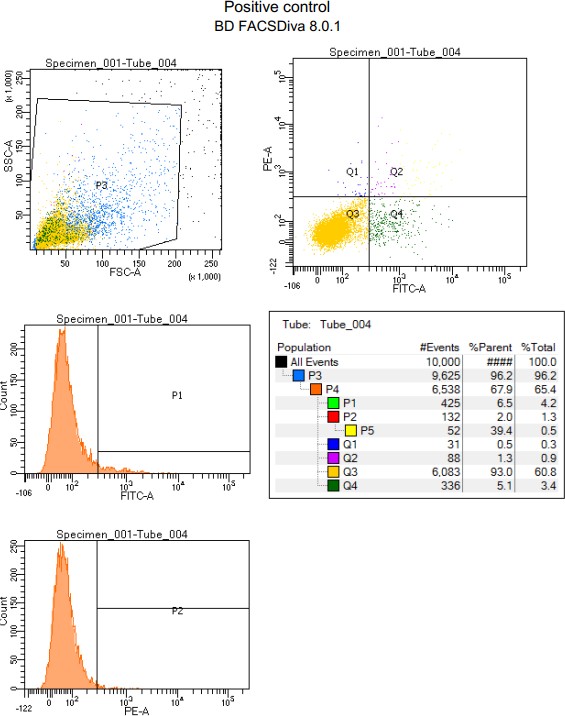


**Repeat.3**


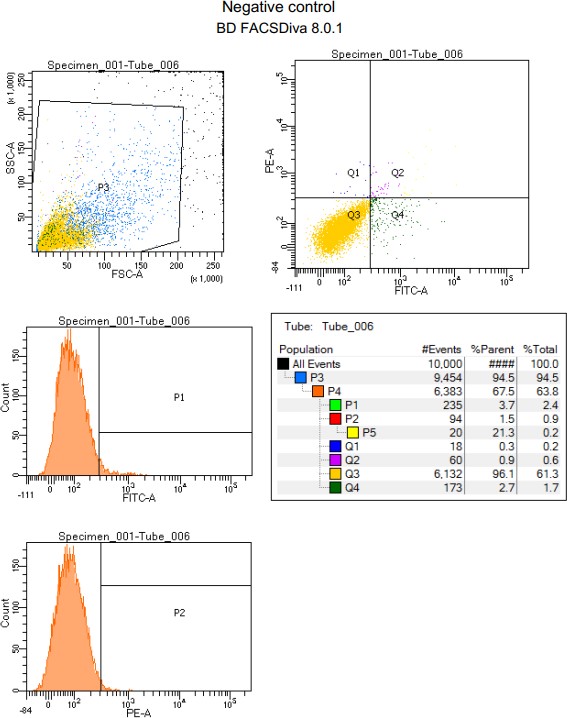


**Repeat.3**


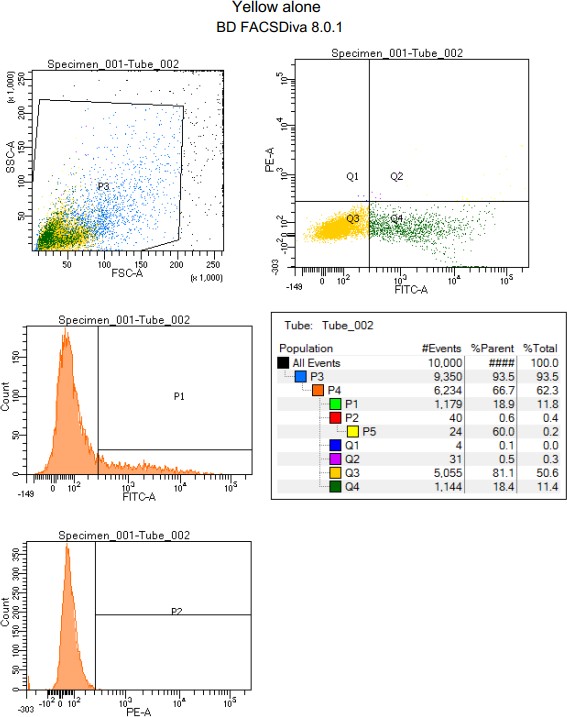


**Repeat.3**


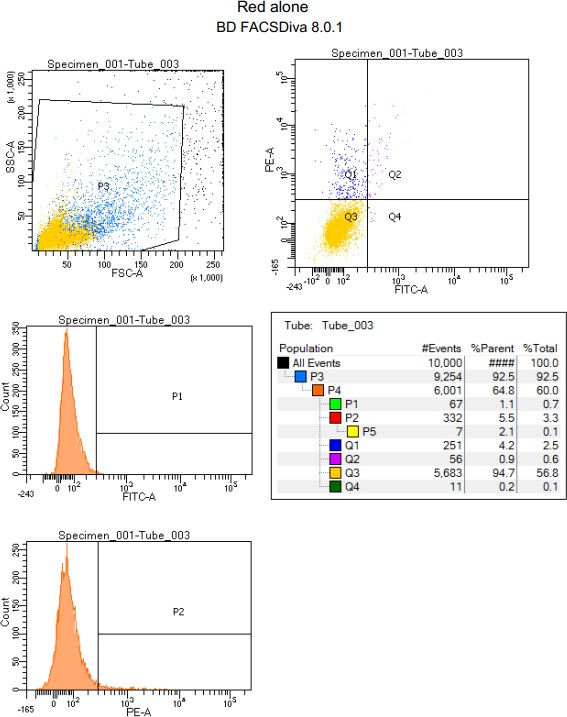


**Repeat.4**


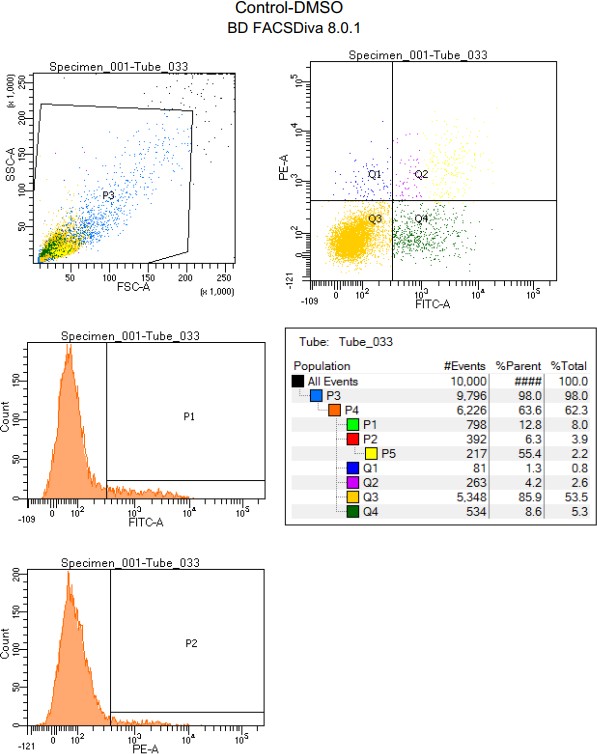


**Repeat.4**


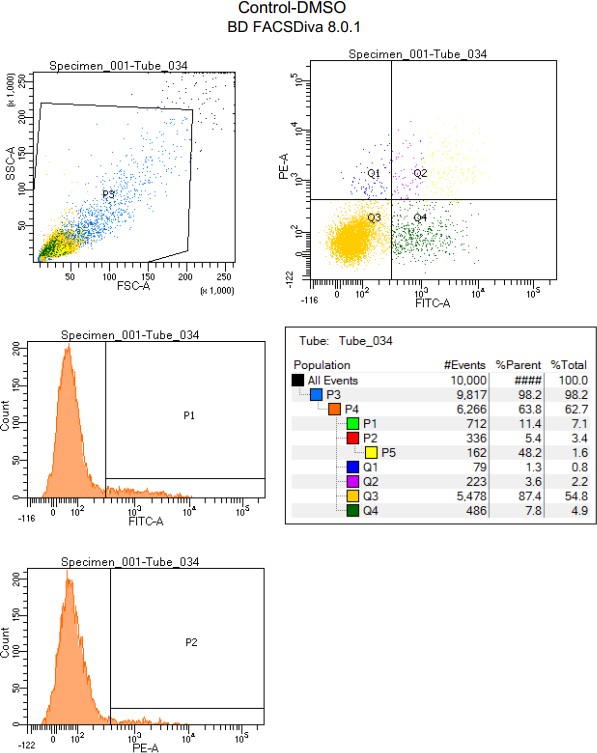


**Repeat.4**


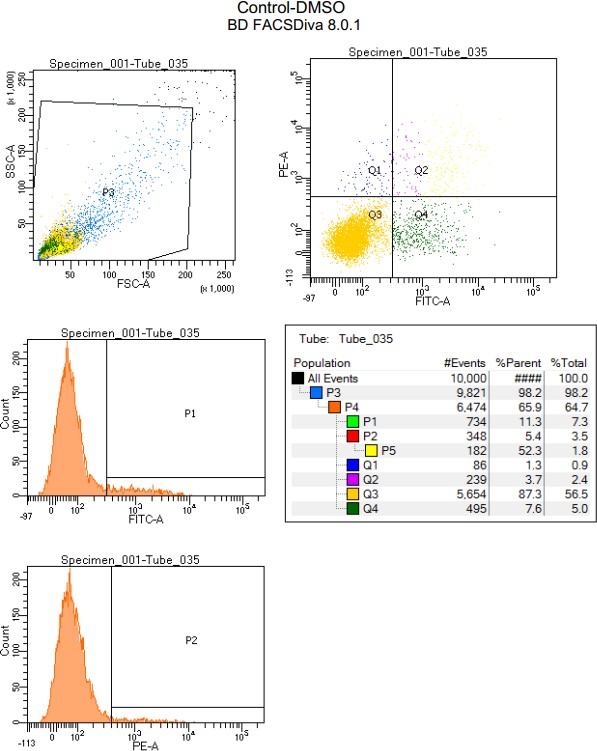


**Repeat.4**


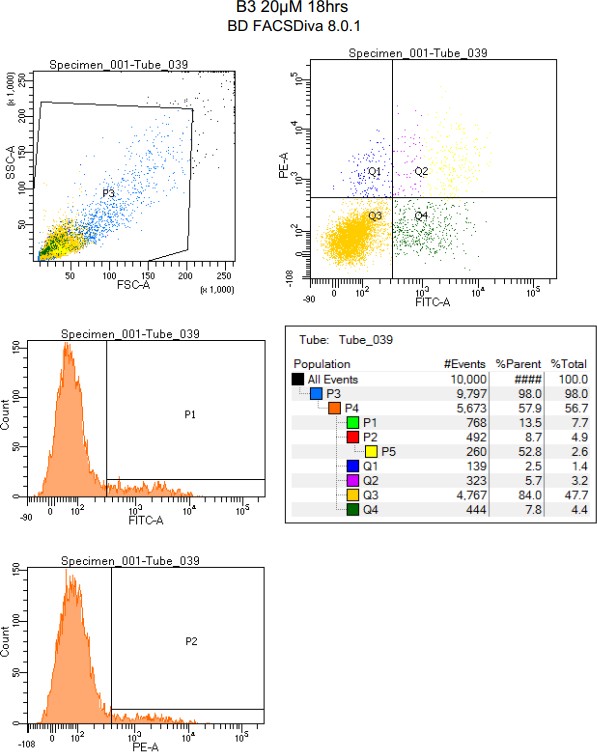


**Repeat.4**


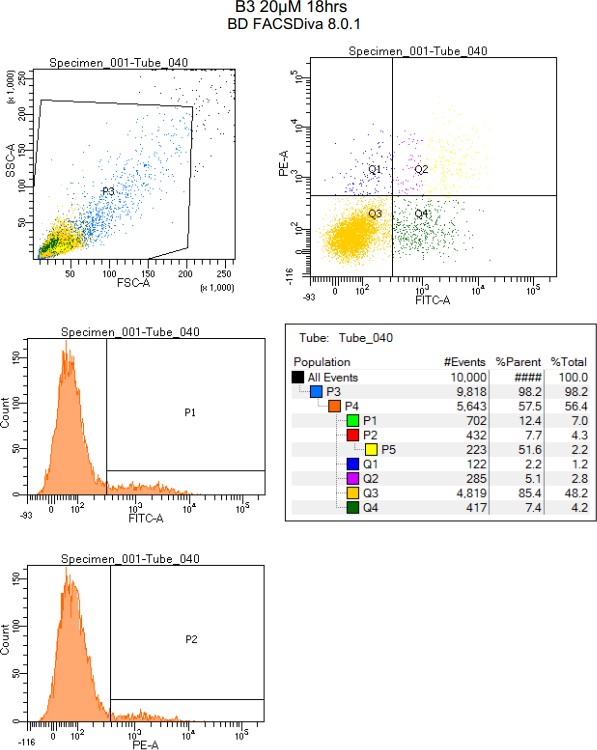


**Repeat.4**


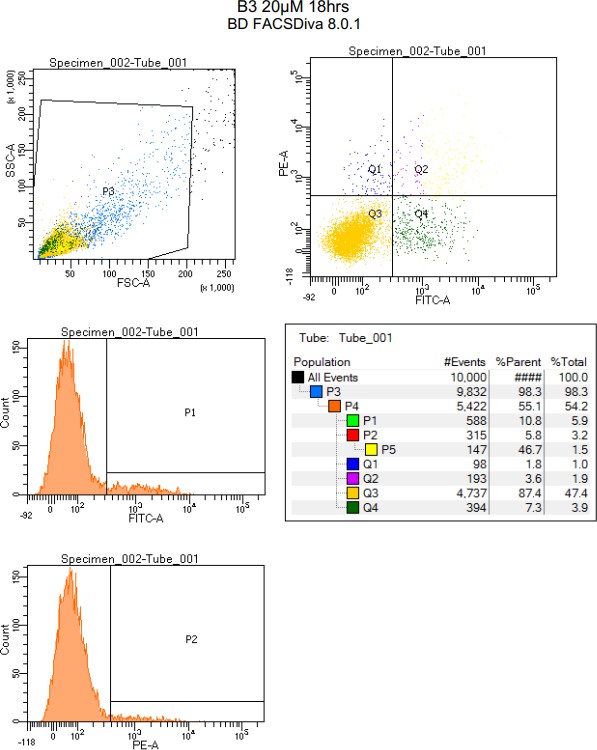


**Repeat.4**


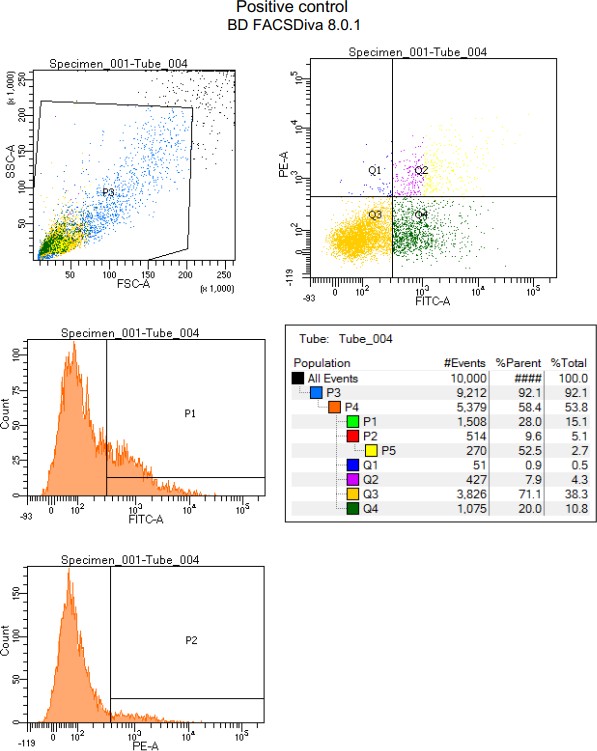


**Repeat.4**


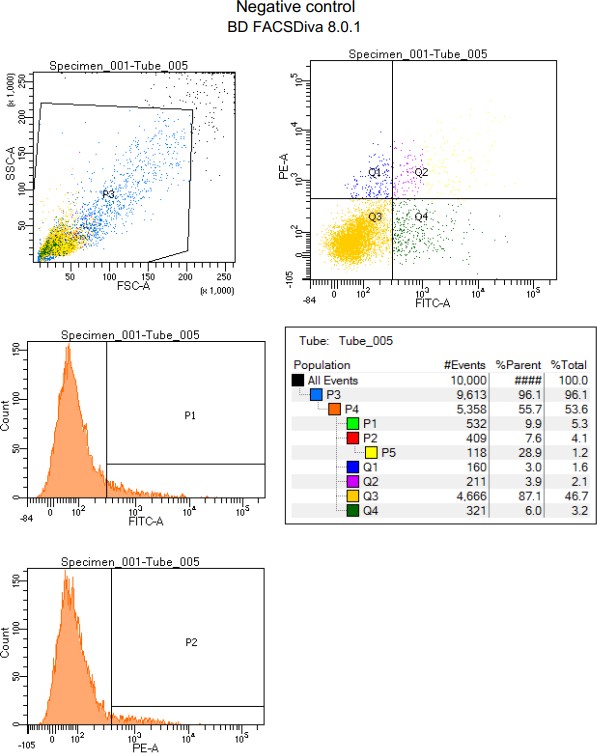


**Repeat.4**


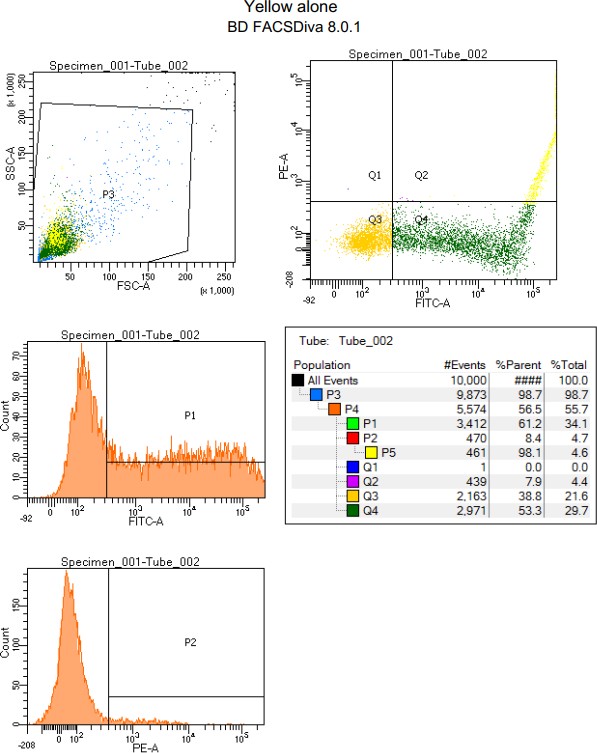


**Repeat.4**


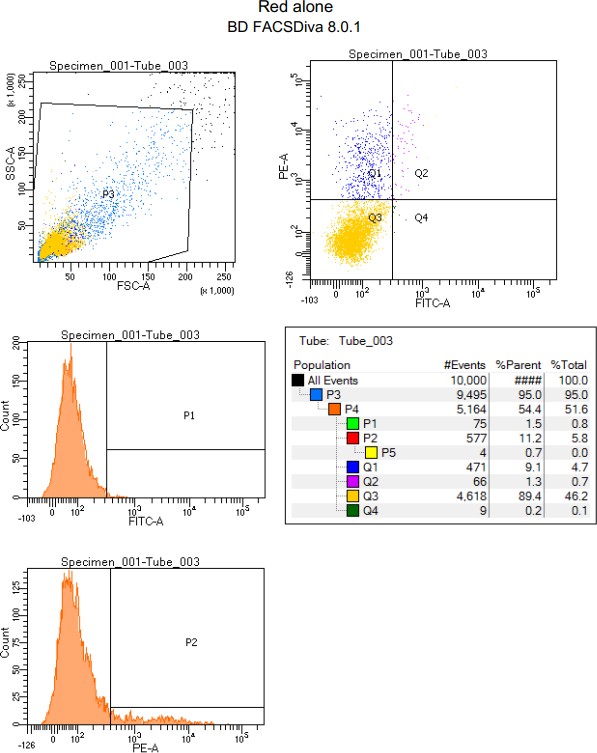

Supplement: Supplementary file 1 — Supplementary file1 (DOCX 6713 KB) [file 10495_2024_1957_MOESM1_ESM.docx]
